# Celastrol induces ROS-mediated apoptosis via directly targeting peroxiredoxin-2 in gastric cancer cells

**DOI:** 10.7150/thno.46728

**Published:** 2020-08-15

**Authors:** Xi Chen, Ying Zhao, Wu Luo, Sian Chen, Feng Lin, Xi Zhang, Shijie Fan, Xian Shen, Yi Wang, Guang Liang

**Affiliations:** 1 Chemical Biology Research Center, School of Pharmaceutical Science, Wenzhou Medical University, Wenzhou, Zhejiang, 325035, China.; 2 Department of Gastrointestinal Surgery, the Second Affiliated Hospital of Wenzhou Medical University, Wenzhou, Zhejiang, 325000, China.; 3 Department of Gynaecology, the First Affiliated Hospital of Wenzhou Medical University, Wenzhou, Zhejiang, 325035, China.; 4 The Affiliated Yueqing Hospital, Wenzhou Medical University, Wenzhou, Zhejiang, 325600, China.

**Keywords:** Peroxiredoxin 2, reactive oxygen species, Celastrol, gastric cancer, oxidative stress

## Abstract

**Background:** Oxidative stress from elevated reactive oxygen species (ROS) has been reported to induce cell apoptosis and may provide a means to target cancer cells. Celastrol is a natural bioactive compound that was recently shown to increase ROS levels and cause apoptosis in cancer cells. However, the underlying mechanism for this cytotoxic action remains unclear and direct molecular targets of Celastrol have not been identified.

**Methods:** Proteome microarray, surface plasmon resonance, isothermal titration calorimetry and molecular simulation were used to identify the molecular target of Celastrol. Binding and activity assays were used to validate the interaction of Celastrol with target protein in cell-free and gastric cancer cell lysates. We then assessed target transcript levels in in biopsy specimens obtained from patients with gastric cancer. Gastric cancer growth-limiting and cytotoxic activity of Celastrol was evaluated in BALB/c nu/nu mice.

**Results:** Our data show that Celastrol directly binds to an antioxidant enzyme, peroxiredoxin-2 (Prdx2), which then inhibits its enzyme activity at both molecular and cellular level. Inhibition of Prdx2 by Celastrol increased cellular ROS levels and led to ROS-dependent endoplasmic reticulum stress, mitochondrial dysfunction, and apoptosis in gastric cancer cells. Functional tests demonstrated that Celastrol limits gastric cancer cells, at least in part, through targeting Prdx2. Celastrol treatment of mice implanted with gastric cancer cells also inhibited tumor growth, associated with Prdx2 inhibition and increased ROS. Analysis of human gastric cancer also showed increased Prdx2 levels and correlation with survival.

**Conclusion:** Our studies have uncovered a potential Celastrol-interacting protein Prdx2 and a ROS-dependent mechanism of its action. The findings also highlight Prdx2 as a potential target for the treatment of gastric cancer.

## Introduction

Targeting biochemical alterations uniquely exhibited by cancer cells may provide therapeutic activity and selectivity in treating cancers 1. Reactive oxygen species (ROS) are normal by-products of cellular processes, such as mitochondrial metabolism and protein folding. Unlike normal cells in which ROS play a role in cell signaling and excess is removed by the antioxidant systems, cancer cells appear to benefit from basally elevated levels of ROS. Slightly elevated ROS in cancer cells may activate proliferation and survival pathways and some oncogenes may also enhance ROS through NADPH oxidases 2, 3. Although various stimuli can induce ROS in cancer cells; excessive levels are, similar to normal cell, kept in check by antioxidant proteins such as peroxiredoxins (Prdx), superoxide dismutase (SOD), catalase, thioredoxin reductase (TrxR), and nuclear factor erythroid 2-related factor 2 (Nrf2). Cancer cells also exhibit increased levels of antioxidant proteins to detoxify ROS.

Increasing ROS in cancer cells that overwhelms the antioxidant clearance has been reported to induce apoptosis through a series of downstream pathways, such as endoplasmic reticulum (ER) stress and mitochondrial cascade 4. Several drugs with cancer-targeting properties such as trisenox 5, paclitaxel 6, and 2-methoxyestradiol 7 have been shown to increase ROS levels. Based on these findings, drugs that regulate cellular redox proteins may offer avenues for cancer treatment. Prdx proteins (Prdx 1-6) are abundant thiol-dependent peroxidases that reduce hydrogen peroxide, peroxynitrite, and other hydroperoxides 8-10. The peroxidase cycle of Prdx proteins is completed through reduction by thioredoxin (Trx)/Trx reductase/NADPH or glutaredoxin systems 11. Typical 2-Cys Prdx such as Prdx2 are susceptible to overoxidation under conditions of high peroxide concentrations, resulting in a Cys sulfonic acid to cause irreversible inactivation of the enzyme 12, 13. Most Prdx family members are increased in various carcinomas and may serve as biomarkers 14, 15. Therefore, identification of new small-molecule inhibitors of Prdx may provide new targets and candidates for cancer treatment.

Celastrol is a bioactive constituent extracted from Tripterygium wilfordii, a traditional Chinese medicinal herb 16-18. Celastrol exhibits a multitude of biological activity. Studies have shown that Celastrol inhibits the proliferation of a wide-range of human tumor cell types, including breast cancer, glioma, head and neck carcinoma, hepatocellular carcinoma, gastric cancer, melanoma, non-small cell lung carcinoma, and prostate cancer 16. Although the mechanisms underlying this inhibitory activity are not fully clear, recent studies found that Celastrol increased ROS levels several-fold and caused cell cycle arrest, apoptosis, and autophagy in cancer cells 19, 20. However, to date, how Celastrol increases cellular ROS and induces cancer cell apoptosis remains unclear.

In this study, we have investigated the mechanism by which Celastrol activates cellular ROS and mitigates the growth of gastric cancer. We found that Celastrol directly binds to and inhibits the activity of Prdx2, resulting in increased ROS levels and apoptotic death in gastric cancer cells. In both culture and mouse studies, Celastrol afforded gastric cancer cytotoxicity through Prdx2 and ROS modulation. We also found that Prdx2 mRNA and protein levels were elevated in human gastric cancer biopsy specimens. Therefore, our studies discovered a precise target of Celastrol and highlighted the importance of targeting Prdx2 in gastric cancer treatment.

## Methods

### General reagents

Celastrol and cisplatin were purchased from Aladdin Chemicals (Shanghai, China). Biotinylated-Celastrol and free-biotin were purchased from Bocong Biotech (Guangzhou, China). Celastrol was dissolved in 0.1% DMSO, and 0.1% DMSO was used as the vehicle control. Annexin V Apoptosis Detection Kit I and propidium iodide (PI) were purchased from BD Pharmingen (Franklin Lakes, NJ). ROS probes 2',7'-dichlorodihydrofluorescein diacetate (DCFH-DA) and dihydroethidium (DHE) were obtained from Beyotime Biotech (Nantong, China). N-acetyl-L-cysteine (NAC), hydrogen peroxide, and puramycin were purchased from Sigma-Aldrich (St. Louis, MO, USA). Malondialdehyde (MDA) levels were measured by using Lipid Peroxidation MDA assay kit (Beyotime Institute of Biotechnology).

Antibodies against phosphorylated-protein kinase R (PKR)-like endoplasmic reticulum kinase (p-PERK), B cell lymphoma-2 (Bcl-2), Bcl-2-associated x protein (Bax), cell proliferation marker Ki67 and GAPDH were purchased from Santa Cruz Biotechnology (Santa Cruz, CA, USA). Antibodies against activating transcription factor-4 (ATF-4), eukaryotic initiation factor 2 (eIF2α, and phosphorylated (p)-EIF2α), CCAAT/enhancer-binding protein homologous protein (CHOP), c-Jun N terminal kinase (JNK and p-JNK), and cleaved-caspase3 were purchased from Cell Signaling Technology (Danvers, MA). Prdx2 antibody was purchased from Abcam. Horseradish peroxidase (HRP)-conjugated secondary antibodies were obtained from Santa Cruz Biotechnology.

### Gastric cancer cell lines

Human gastric cancer cell lines SGC-7901 and BGC-823 were purchased from the Institute of Biochemistry and Cell Biology, Chinese Academy of Sciences (China). Cells were cultured in RPMI-1640 medium (Gibco, Eggenstein, Germany) supplemented with 10% heat-inactivated fetal bovine serum (Gibco), 100 U/mL penicillin and 100 μg/mL streptomycin. Cells were treated with Celastrol at various concentrations and for different time periods as indicated. NAC pretreatment, where indicated, was carried out at 5 mM for 1 h.

### Cell viability and apoptosis assays

To measure viability of cells following exposure to Celastrol, cells were seeded at a density of 6 × 10^3^ per well in 96-well plates and allowed to attach overnight. Cells were then exposed to Celastrol at various concentrations for 24 or 48 h. For some experiments, free biotin or biotinylated-Celastrol were used. Following treatments, 0.5 mg/mL MTT reagent was added through fresh media and cells were cultured for an additional 4 h. DMSO was used to dissolve the formazan product and absorbance was measured at 490 nm using SpectraMax M5 microplate reader (Molecular Devices, USA). The percent inhibition rate was calculated as: [1 - (treated/ control)] × 100. The IC_50_ values were obtained using the Logit method.

For apoptosis determination, SGC-7901 and BGC-823 were plated at 3 × 10^5^ cells per well in 6-well culture dishes for 24 h. Cells were then exposed to Celastrol for 24 h and stained with FITC-conjugated Annexin V and PI. Data were collected and analyzed using FACSCalibur flow cytometer. Phase contrast images of cells treated as indicated above were also captured using Nikon epifluorescence microscope (Nikon, Japan). In addition, fluorescence images were captured following staining of cells with PI. DAPI was used as counterstain for images.

Caspase-3 and 9 activity in cell lysates was determined using a Caspase-3/9 Activity kits (Beyotime Institute of Biotechnology, Nantong, China). Activity levels were normalized to total protein levels of the corresponding cell pellet and expressed as percentage of treated cells to that of control.

### Electron microscopy

SGC-7901 cells were cultured in vehicle control (DMSO) or 3 µM Celastrol for 8 h. NAC pretreatment where indicated was carried out for 1 h. Cells were then fixed with 2.5% glutaraldehyde overnight at 4 °C. Cells were post-fixed in 1% OsO4 at room temperature for 60 min, stained with 1% uranyl acetate, dehydrated through graded acetone solutions, and embedded in epon. Areas containing cells were block-mounted and cut into 70 nm sections and examined with an electron microscope (H-7500, Hitachi, Ibaraki, Japan).

### Expression and purification of Prdx2 and Prdx2 mutants

His-tagged Prdx2 in pET28a vectors was used to transform *E. coli* BL21. Cells were grown with 50 g/ml kanamycin at 37 °C to an absorbance of 0.6 at 600 nm. Expression was induced with 0.25 mM Isopropyl-D-1-thiogalactopyranoside (IPTG) for 12 h at 20 °C. Cell pellets were prepared and suspended in lysis buffer (50 mM NaH_2_PO_4_ and 0.3 M NaCl, pH 8.0) and disrupted by high pressure homogenizer. After centrifugation (12,000 xg for 30 min at 4 °C), the supernatant incorporated His-tagged recombinant Prdx2 (rhPrdx2) protein was collected and applied onto purification by AKTA purifier system using a 5 mL HisTrap^TM^ Chelating column (GE Healthcare, USA). The column was washed with 100 mL of binding buffer (50 mM NaH_2_PO_4_ and 0.3 M NaCl, pH 8.0), and rhPrdx2 protein was eluted (50 mM NaH_2_PO_4_, 0.3 M NaCl, and 200 mM Imidazole, pH 8.0).

To generate Prdx2 Cys172 to Ser172 (C172S) and Prdx2 Cys172 to Ala172 (C172A) variants, site-directed mutations were carried out using QuikChange site-directed mutagenesis kit (Stratagene, Palo Alto, CA). The specific primers used for the point mutations of human Prdx2 genes are listed in Table S4.

### Peroxiredoxin activity assays

Peroxiredoxin (Prdx) activity was determined as described previously 21, 22. Recombinant Prdx2 proteins either alone or mixed with different concentrations of Celastrol were incubated with 50 mM Hepes-HCl (pH 7.0) containing 1 mM DTT for 10 min at room temperature. The reaction was initiated by the addition of 1 μL H_2_O_2_ (final concentration 50 μM) and incubated for 5 min. The residual H_2_O_2_ amount was measured. The reaction mixture contained 4 μM/L Amplex Red and 1 U horseradish peroxidase. The remaining peroxide content was determined by measuring emission at 590 nm. Adenanthin was used as positive control 23. Prdx activity was measured similarly in cell lysates.

### Colony formation assay

Cells were seeded at 500 cells per well in 6-well plates and cultured in the presence of Celasterol for 8 h. Cells were allowed to grow for 8 days in normal growth media and stained with crystal violet solution (0.5 in 25% methanol) to assess colony growth. A colony is defined as a cluster of at least 50 cells that can often only be determined microscopically.

### Measurement of ROS generation in cells

Cellular ROS generation was measured by flow cytometry utilizing a ROS-sensitive dye. Briefly, 3×10^5^ cells were plated in 6-well culture dishes and cultured overnight in normal growth media. Cells were then challenged with Celastrol at concentrations and times indicated. Cells were stained with 10 μM DCFH-DA at 37 °C for 30 min. ROS levels were measured by flow cytometry (FACSCalibur, BD Biosciences, CA). In some experiments, cells were pretreated with 5 mM NAC for 1 h. In all experiments, 8000 viable cells were analyzed.

### Determination of mitochondrial membrane potential

Cells were cultured as indicated and mitochondrial membrane potential was determined by cell permeable JC-1 dye. JC-1 selectively enters the mitochondria and reversibly changes from green to red as the membrane potential increases. In healthy cells with normal ΔψM, JC-1 spontaneously forms J-aggregates with intense red fluorescence. Low ΔψM in apoptotic cells maintains JC-1 in the monomeric form and only shows green fluorescence. JC-1 fluorescence images of cells exposed to Celastrol, with or without NAC pretreatment, were captured using an epifluorescence Nikon microscope (Nikon, Japan).

### Cell transfections for gene silencing and expression

The Prdx2 siRNA duplexes used in this study were purchased from Invitrogen (Carlsbad, CA) and have the following sequence: 5'-UUAGGCUGGCUAACGGAUA-3'. Negative Control (NC) was used as control. BGC-823 cells were seeded at 2 × 10^5^/well on 6-well plates and cultured for 24 h. Cells were transfected with siRNA duplexes by lipofectamine 3000 (Invitrogen). Following transfection, cells were challenged with Celastrol for 24 h before apoptosis detection. For some experiments, we knocked down Prdx2 by transfecting cells with shRNA expressing plasmids (pGV287-GFP-shRNA-Prdx2; Shanghai GeneChem Co) with pylobrene (6 μg/mL) (Thermo Fisher). For these studies, pGV287-GFP plasmid was used as negative control. Cells expressing the plasmid were purified by culturing in 2 µg/mL puromycin. Prdx2 overexpression was carried out with Prdx2 cDNA plasmid (pGV492-GFP-O/E-Prdx2; Shanghai GeneChem Co) and using the same protocol as indicated.

### Proteome microarray assays

Arrayit HuProt™ v2.0 19K Human Proteome Microarrays (CDI Laboratories, Baltimore, MD) were used. Microarrays were incubated with 50 µM biotinylated-celastrol or free-biotin. After washing, Cy3-Streptavidin (1:1000, Sigma-Aldrich, St Louis, MO) was added. Genepix 4200A (Axon Instruments, Sunnyvale, CA) was used for scanning. Data were analyzed by GenePix Pro 6.0.

### Molecular docking of Celastrol to Prdx2

The crystal structure of Prxd2 was derived from Protein Data Bank (PDB code: 5IJT). The flexible loop containing residue Cys172 was modeled by Modeller 24. Then, the AutoDock software was used to dock Celastrol into the dimerization pocket using default parameters 25.

### Surface Plasmon Resonance (SPR) analysis

The binding affinity of Celastrol to recombinant human Prdx1 and Prdx2 was determined at 22 °C using Biacore T200 instrument equipped with CM5 sensor chips (GE healthcare). Briefly, rhPrdx1 and 2 proteins in acetate acid buffer (pH 5.5) were immobilized on the sensor after activation by 40 mM EDC and 10 mM NHS in water solution. Different concentrations of Celastrol, including 100, 50, 25, 12.5, 6.25 and 0 μM were prepared with running buffer (phosphate-buffered saline, 0.1% sodium dodecyl sulfate and 0.05% Tween-20). The compounds at different concentrations were injected simultaneously. Data processing and analysis were performed using Biacore 4000 and Biacore T200 evaluation software (GE healthcare).

### Celastrol-Prdx binding assays

The binding activity of Celastrol to recombinant Prdx2 was examined by the isothermal titration calorimetry (ITC) assay using auto-iTC200 instrument (MicroCal, GE). Recombinant Prdx2 was loaded into a 96-well plate, and then Celastrol was titrated using a syringe. The equilibrium time between two adjacent injections was 210 s. The binding stoichiometry (n), binding constant (Kd) and thermodynamic parameters (ΔH and ΔS) were determined by fitting the titration curve to a one-site binding mode, using the Origin software provided by the manufacturer.

We also utilized Pierce^TM^ Biotinylated Protein Interaction Pull-Down (Thermo Fisher) to examine Celastrol-Prdx2 interaction. One hundred µL of 20 mM biotinylated-Celastrol was added to 50 μL streptavidin-agarose beads and incubated at 4 °C for 30 minutes. Biotin alone was used as a control. Lysates prepared from gastric cancer cells, cells transfected with Prdx2 variants, and tumor tissues were then added to the streptavidin-agarose beads with bio-Celastrol. The mixture was incubated at 4 °C for 24 hours with gentle rocking. Samples were then spun and washed 3 times. Elution Buffer was added into each spin column. Eluent was boiled with 5× loading buffer, and the samples were loaded on a 10% polyacrylamide gel for Western Blot analysis. Total lysates were used as an input control.

### Real-time quantitative PCR

RNA was isolated from cultured cells and tumor tissues using TRIZOL (Thermo Fisher). Reverse transcription and quantitative PCR was carried out using two-step M-MLV Platinum SYBR Green qPCR SuperMix-UDG kit (Thermo Fisher) and Eppendorf Mastercycler ep realplex detection system (Eppendorf, Hamburg, Germany). Primers for genes were synthesized and obtained from Thermo Fisher. The sequences of Prdx2 and housekeeping gene primer are listed in Table S4. Prdx2 mRNA data was normalized to β-actin housekeeping gene.

### Western immunoblot analysis

Fifty micrograms of cell and tissue lysates were separated by 10% SDS-PAGE and electro-transferred to a PVDF membranes. Membranes were blocked in Tris-buffered saline containing 0.05% Tween20 and 5% non-fat milk for 1.5 h. PVDF membranes were then incubated with specific primary antibodies. Immunoreactive bands were detected by incubating with a secondary antibody conjugated with horseradish peroxidase and enhanced chemiluminescence reagent (Bio-Rad). The amounts of the proteins were analyzed using Image J version 1.38e and normalized to their respective control.

### Patient samples

This study was approved by the Institutional Research Human Ethical Committee of the Wenzhou Medical University for the use of clinical biopsy specimens and informed consent was obtained from patients. A total of 17 gastric cancer biopsy samples were obtained. Clinical diagnosed was performed at the Second Affiliated Hospital of Wenzhou Medical University during 2017-2018. Frozen gastric cancer specimens and patient-matched, tumor-adjacent, morphologically normal gastric tissues were used. Total RNA and proteins were extracted and subjected to qPCR and western blotting. Patient information is provided in the Table S3. To correlate Prdx2 with patient survival, data was obtained from a database at http://kmplot.com/analysis/index.php?p=service. Prdx2 probes (201006_at, cutoff value 145; 215067_x_at, cutoff value 265) were used to assess survival with Prdx2 expression.

### *In vivo* xenograft model

All animal experiments complied with the Wenzhou Medical University's Policy on the Care and Use of Laboratory Animals. Protocols for animal studies were approved by the Wenzhou Medical College Animal Policy and Welfare Committee and followed the National Institutes of Health guide for the care and use of Laboratory animals (NIH Publications No. 8023, revised 1978). Protocols were also in compliance with ARRIVE guidelines 26. Five-week-old, athymic BALB/c *nu/nu* female mice were purchased from Vital River Laboratories (Beijing, China). Mice were housed at a constant room temperature with a 12:12 h light/dark cycle and fed a standard rodent diet and given water *ad lib*. Mice were randomly divided into three experimental groups (n=6): control, Celastrol 1, and Celastrol 2. SGC-7901 cells were injected subcutaneously into the right flank of all mice at 1 × 10^7^ cells in 150 μL PBS. After day 10 of cell injection, treatment of mice was initiated. All mice showed tumors reaching 50-100 mm^3^ volume at this starting point. Treatments included Celastrol at 1 or 2 mg/kg every other day by intraperitoneal (ip) injections. Control mice received PBS vehicle control. Tumor volumes were determined by measuring length (*l*) and width (*w*) and calculating volume (*V* = 0.5 × *l* × *w*^2^) at the indicated time points. Treatments were maintained for 24 days. Mice were sacrificed, and tumor tissues were removed, weighed, and photographed. Samples were used for histology and proteins level determination.

To assess the involvement of oxidative stress in tumor tissues, mice were randomly divided into four experimental groups (n=6): Control, Celastrol, NAC+Celastrol, and Cisplatin (positive control). SGC-7901 tumors were produced as described above. Control mice received PBS. Celastrol treatment was carried out with 1.5 mg/kg administered every other day. For the NAC+Celastrol group, NAC was provided at 1 g/L in drinking water for the duration of the study. Cisplatin was used as positive control and administered at a dose of 5 mg/kg every other day (PBS with 6% castor oil).

To confirm the involvement of Prdx2, we tested the effect of Celastrol in gastric cancer cells with Prdx2 overexpression or knockdown. Specifically, BGC-823 cells which express Prdx2 robustly were transfected with Prdx2 shRNA and used to generate tumors. Conversely, SGC-7901 cells were transfected with Prdx2 cDNA plasmids and injected in mice. Mice harboring BGC-823 and SGC-7901 tumors were divided in four experimental groups each (n = 6). BGC-823 xenografts included: control shRNA (untreated mice), Prdx2 shRNA (untreated mice), Control shRNA + Celastrol, and Prdx2 shRNA with Celastrol treatment. Celastrol treatments were initiated after day 8 of cell injection. Celastrol treatment was carried out at 1.5 mg/kg every other day. Untreated mice received PBS vehicle control. Similar groupings were performed with mice grafted with SGC-7901 cells.

### 3D *in vitro* model of human gastric cancer

To generate a 3D *in vitro* model of gastric cancer, harvested tissues were washed with PBS and cut into pieces of 0.8-1.2 mm in diameter. Tumors were transferred into 6 well plates (4-6 tissues for each well and 6 wells for each group). Each well was maintained with 1 mL culture medium. The full medium was supplemented with Advanced DMEM/F12 (Gibco, life technologies), B-27 (50x, Gibco) and Antibiotic-Antimycotic solution (Gibco). Tissues were cultured in a humidified incubator at 37 ℃ and 5% CO_2_.

### Tissue immunostaining

For immunohistochemistry, tumor specimens were fixed in 4% paraformaldehyde and embedded in paraffin. Five μm thick sections were stained with Ki-67 to assess tumor cell proliferation and cleaved-caspase 3 for apoptosis. Briefly, heat-induced antigen retrieval was performed using 10 mM sodium citrate buffer, pH 6.5. Peroxidase activity was quenched with 3% H_2_O_2_ and tissues were blocked in 5% bovine serum albumin for 30 min. Primary Ki-67 (1:200) and cleaved-caspase 3 antibody (1:100) was added and sections were incubated overnight at 4 °C. Horseradish peroxidase-conjugated secondary antibody (1:1000) and DAB were used for detection. Slides were counterstained with hematoxylin.

Frozen tumor tissue sections were used for immunofluorescence staining for ROS. Sections were washed and incubated with dihydroethidium (DHE) or DCFH-DA. Images were captured with epifluorescence microscope.

### Statistical analysis

All experiments are randomized and blinded. Data from 3 independent experiments is expressed as Mean ± SEM. The exact group size (n) for each experimental group/condition is provided and 'n' refers to independent values, not replicates. Statistical analysis was performed with GraphPad Prism 6.0 software (San Diego, CA, USA). We used one-way ANOVA followed by Dunnett's post hoc test when comparing more than two groups of data and one-way ANOVA, non-parametric Kruskal-Wallis test, followed by Dunn's post hoc test when comparing multiple independent groups. *P* values of < 0.05 were considered statistically significant. Post-tests were run only if F achieved *P*< 0.05 and there was no significant variance in data homogeneity.

## Results

### Celastrol induces apoptosis by increasing ROS in gastric cancer cells

**Figure 1:**
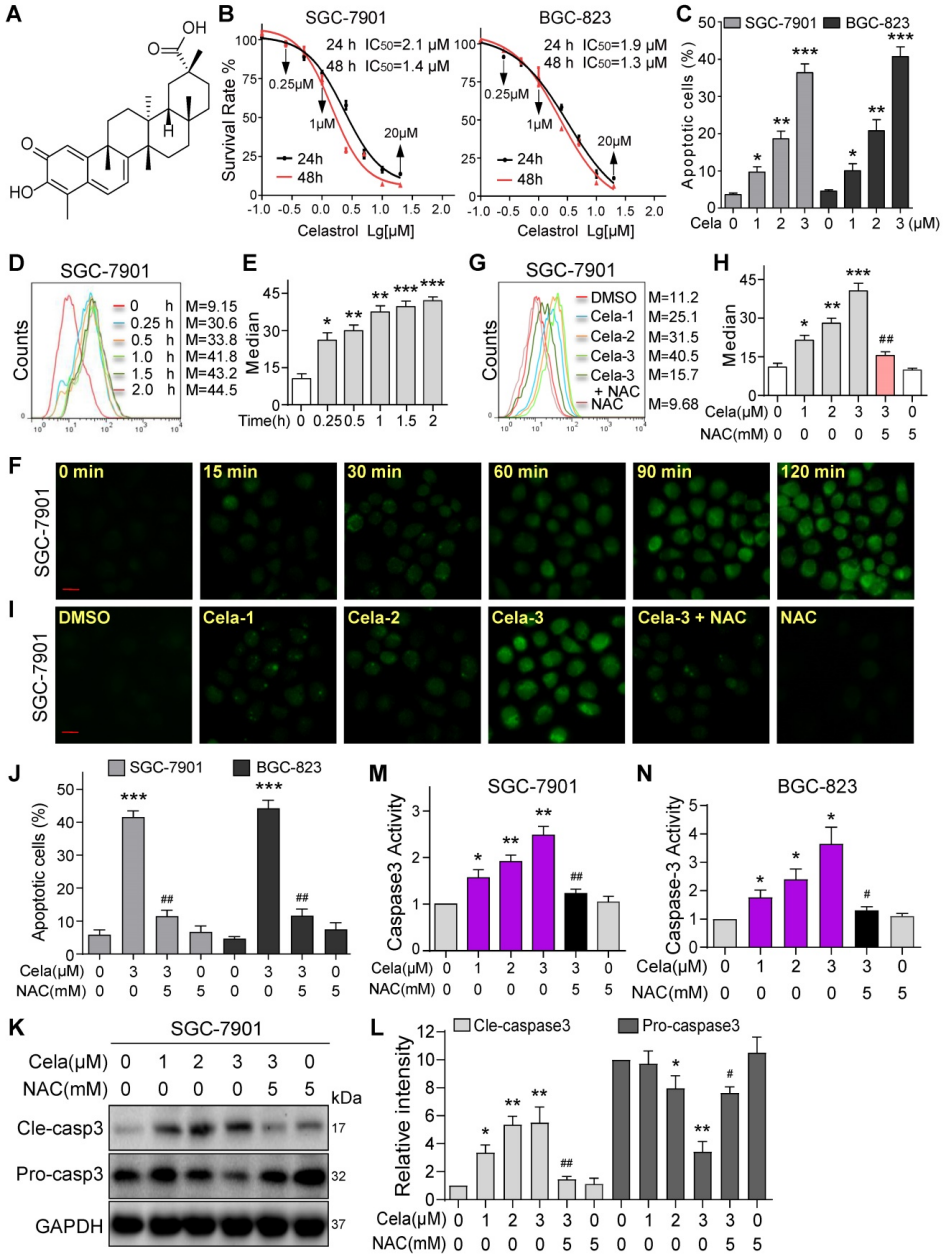
** Celastrol induces apoptosis via increasing ROS in gastric cancer cells.** (**A**) Chemical structure of Celastrol (Cela). (**B**) Effect of Celastrol on the viability of human gastric cancer cells. Cells were challenged with increasing concentrations of Celastrol for 24 or 48 h and cell viability was measured by MTT. IC_50_ values in two different cell lines are shown (n = 4). (**C**) Effect of Celastrol on gastric cancer cell apoptosis as assessed by Annexin V/PI staining. Cells were exposed to Celastrol for 24 h. The percentage of cells shown are double positive for Annexin V and PI (n = 3; *P<0.05, **P<0.01, ***P<0.001 compared to DMSO control). (**D, E**) Intracellular ROS generation was measured by DCFH-DA. SGC-7901 cells were exposed to 3 µM Celastrol for indicated times. Representative flow cytometric graph shown in panel D. Quantification of ROS levels in SGC-7901 cells is shown in panel E (n = 3; *P<0.05, **P< 0.01, ***P<0.001 compared to 0 h control). (**F**) Intracellular ROS as detected by DCFH-DA staining (green). SGC-7901 cells were exposed to 3 µM Celastrol for indicated times and fluorescence images were captured (scale bar = 20 µm). (**G, H**) SGC-7901 cells were challenged with Celastrol at 1, 2, or 3 µM for 3 h. NAC was used at 5 mM, either alone or as a 1 h pretreatment. Representative flow cytometric graph shown in panel G. Quantification of ROS levels in SGC-7901 cells is shown in panel H (n = 3; *P<0.05, **P< 0.01, ***P<0.001 compared to Control; ##*P*<0.01 compared to Cela-3). (**I**) SGC-7901 cells were treated as indicated in panel G and fluorescence images showing DCFH-DA (green) were captured (scale bar = 20 µm). (**J**) Effect of NAC on Celastrol-induced apoptosis. Cells were pretreated with 5 mM NAC for 1 h and then exposed to 3 µM Celastrol for 24 h. The apoptotic cells were assessed by Annexin V/PI staining. The percentage of cells shown are double positive for Annexin V and PI (n = 3; ***P<0.001, compared to DMSO control; ^##^*P*<0.01, compared to Cela alone). (**K, L**) NAC inhibits Celastrol-induced caspase-3 activation. SGC-7901 cells were exposed to Celastrol at indicated concentrations for 20 h, with or without 1 h pretreatment with 5 mM NAC. Panel K shows immunoblots of cleaved-caspase-3 and caspase-3. GAPDH used as loading control. Panel L shows quantification of cleaved-caspase-3 and caspase-3 levels in SGC-7901 cells (n = 3; *P<0.05, **P<0.01 compared to Control; ^#^*P*<0.05, ^##^*P*<0.01 compared to Cela-3). (**M, N**) NAC reduces Celastrol-induced caspase-3 activity. Cells were treated as shown in panels K and L, and caspase-3 activity was measured (n = 3; *P<0.05, **P< 0.01 compared to Control; ^#^*P*<0.05, ^##^*P*<0.01 compared to Cela-3).

We first wanted to confirm the effects of Celastrol on gastric cancer cell viability. To do this, we selected two human gastric cancer cell lines (SGC-7901 and BGC-823) and challenged the cells with increasing concentrations of Celastrol. Celastrol (Figure 1A) inhibited both cancer cell lines with comparable IC_50_ of 1.9-2.1 µM at 24 hours and 1.3-1.4 µM at 48 hours (Figure 1B). Colony formation assay showed reduced growth in SGC-7901 and BGC-823 with increasing concentrations of Celastrol (Figure S1). Furthermore, flow cytometric quantification of apoptotic cells showed Celastrol cytotoxicity (Figure 1C, Figure S2). These results show that Celastrol induces apoptosis in gastric cancer cells. Based on these studies, we selected 1-3 µM Celastrol to probe underlying mechanisms in gastric cancer cells. We examined that if Celastrol induces cell apoptosis by modulating ROS levels. We exposed SGC-7901 and BGC-823 cells to 3 µM Celastrol and measured ROS by DCFH-DA staining. Our data shows that Celastrol significantly increases cellular ROS levels and this induction appears as early as 15 minutes after exposure in both cell lines (Figure 1D-F, Figure S3). Celastrol also showed a dose-dependent induction of ROS in SGC-7901 (Figure 1G-I) and BGC-823 cells (Figure S4). As expected, this ROS signal was diminished in cells pretreated with the antioxidant N-acetyl cysteine (NAC). NAC was then used to determine the involvement of ROS in Celastrol-induced apoptosis in gastric cancer cells. Pretreatment of cells with 5 mM NAC prevented Celastrol-induced apoptosis in SGC-7901 and BGC-823 cells (Figure 1J, Figure S5). These results were confirmed by cleaved caspase 3 levels (Figure 1K, Figure S6), and caspase 3 activity levels (Figure 1M-L). These results show that enhanced ROS levels contribute to cytotoxicity of Celastrol in gastric cancer cells.

### Celastrol activates ER and mitochondrial pathways of cell death through ROS

**Figure 2:**
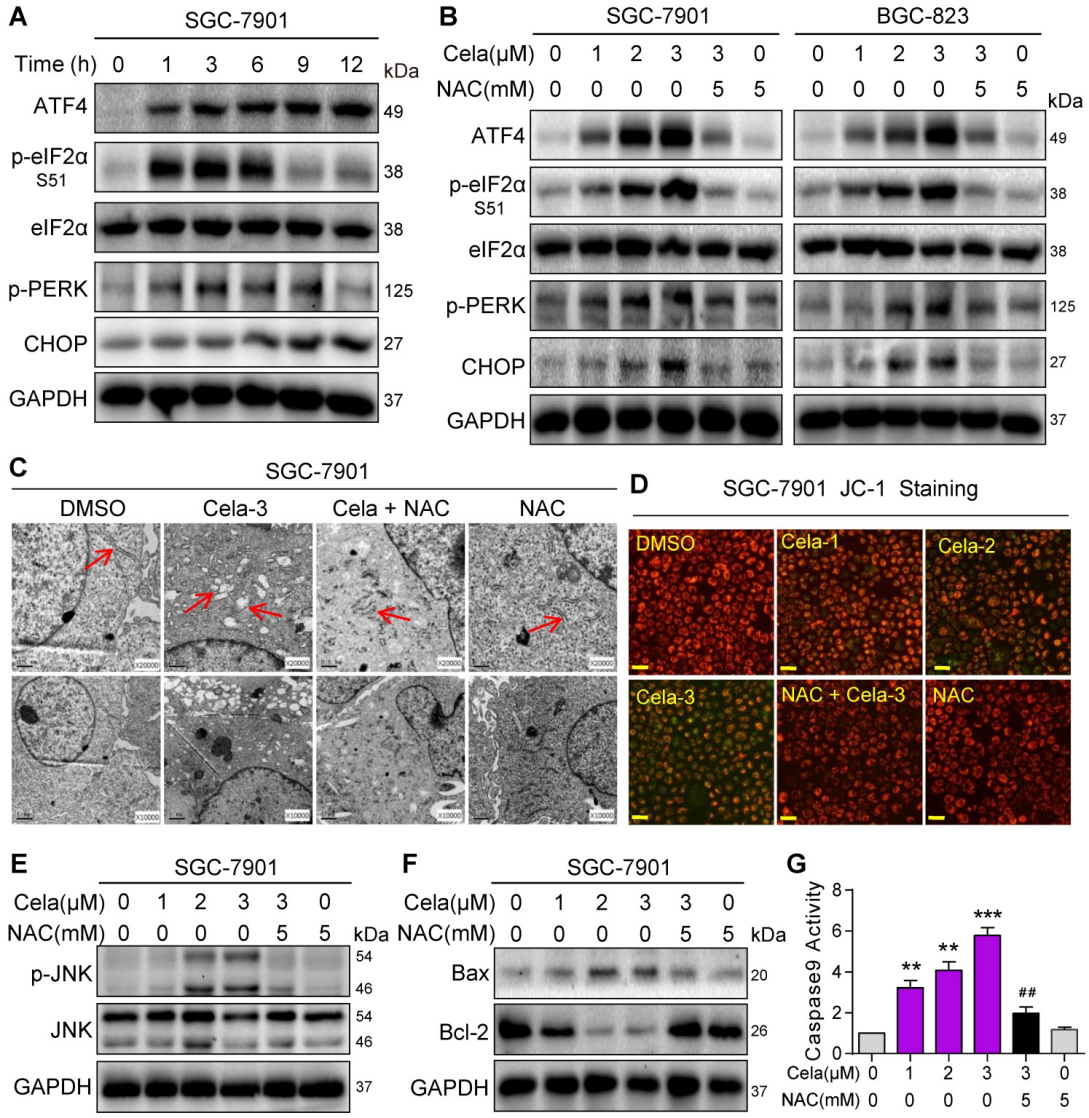
** Celastrol activates pro-apoptotic ER stress and mitochondrial pathways via increasing ROS.** (**A**) Rapid activation of the ER stress pathway by Celastrol. SGC-7901 cells were exposed to 3 µM Celastrol for indicated times. Lysates were probed for p-eIF2α, p-PERK, ATF4, and CHOP. eIF2α and GAPDH served as controls. (**B**) Effect of NAC on Celastrol-induced ER stress. SGC-7901 and BGC-823 Cells were pretreated 5 mM NAC for 1 h where indicated and exposed to Celastrol for 3 h (ATF-4, p-eIF2α, eIF2α, p-PERK) or 8 h (CHOP). Lysates were processed to Western blot assay. (**C**) Electron microscopy images of SGC-7901 cells exposed to 3 µM Celastrol for 8 h. Arrows pointing to ER [images shown are 10000 magnification in lower panel and 20000 magnification in upper panels]. (**D**) Mitochondrial membrane potential (Δψm) was detected by JC-1 dye. SGC-7901 cells were pretreated with 5 mM NAC for 1 h before exposure to 3 µM Celastrol for 12 h [scale bar = 40 µm]. (**E, F**) Western blot analysis of p-JNK/JNK (E) and Bax/Bcl-2 (F) in SGC-7901 cells exposed to Celastrol at indicated concentrations for 12 h. NAC pretreatment was carried out at 5 mM for 1 h. GAPDH was used as loading control. (**G**) NAC inhibits Celastrol-induced caspase-9 activation. SGC-7901 cells were exposed to Celastrol at indicated concentrations for 20 h, with or without 1 h pretreatment with 5 mM NAC. The caspase-9 activity was measured using a substrate kit (n = 3; **P<0.01, ***P<0.001 compared to Control; ^##^*P*<0.01 compared to Cela-3).

Elevated ROS levels have been reported to induce apoptosis by a series of downstream pathways including ER stress and mitochondrial dysfunction 27. We determined whether increased ROS levels by Celastrol induce ER stress in gastric cancer cells. We examined changes in ER stress-associated proteins including ATF4, phosphorylated (p)-eIF2α, p-PERK, and CHOP. These markers of ER stress were rapidly elevated in SGC-901 cells exposed to Celastrol (Figure 2A). NAC pretreatment was able to prevent this induction in SGC-7901 and BGC-823 cells (Figure 2B). Electron microscopy showed that Celastrol causes ER swelling in gastric cancer cells, which is not observed in cells pretreated with NAC (Figure 2C, Figure S7).

We then investigated the changes in mitochondrial membrane potential in cells challenged with Celastrol. Analysis of cells following Celastrol exposure showed a disruption of mitochondrial membrane potential as detected by JC-1 dye (Figure 2D, Figure S8). Similar to ER stress assessment, mitochondrial disruption by Celastrol was not seen when cells were pretreated with NAC indicating that Celastrol-induced ROS was the driver. We know that induction of pro-apoptotic Bax and its association with mitochondrial damage 28, and activation of stress and apoptotic pathways such as JNK 29 and caspase-9 30, 31 play a role in cell apoptosis. We probed these mechanisms in gastric cancer cells and show that Celastrol induces phosphorylation of JNK (Figure 2E, Figure S8) and decreases Bcl-2:Bax ratios in both cell lines (Figure 2F, Figure S9). As anticipated, these alterations were normalized by NAC. We also found that Celastrol-induced caspase-9 activation was normalized by NAC pre-treatment in cells (Figure 2G, Figure S11). These findings provide strong evidence that Celastrol induces ROS, leads to ER stress and mitochondrial dysfunction, and initiates apoptosis in gastric cancer cells.

### Celastrol directly binds and inhibits peroxiredoxins

**Figure 3:**
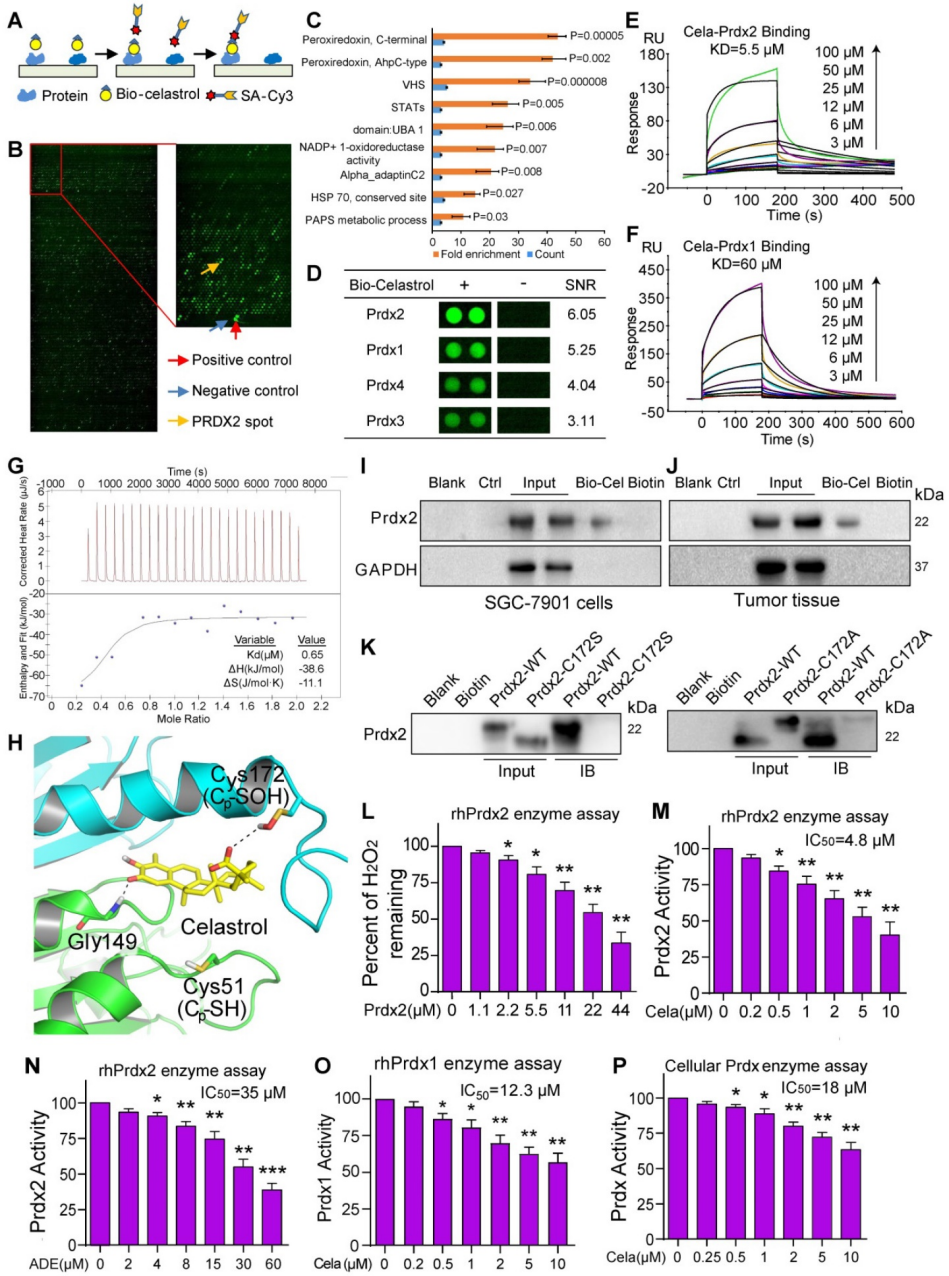
** Celastrol directly binds to Prdx2 and inhibits its activity.** (**A**) Schematic of the procedure for detecting Celastrol-binding proteins. (**B**) Human protein microarrays were probed with biotinylated-Celastrol. Binding was detected by Cy3-labeled streptavidin. Control experiments were carried out with free biotin (green pots showing interacting proteins). (**C**) Pathway enrichment analysis by DAVID. Celastrol showed the strongest binding ability to Prdx proteins. (**D**) Representative Prdx signals from the protein arrays. Signal-to-noise ratios (SNR) are shown (- = free biotin). (**E, F**) The binding affinity of Celastrol with rhPrdx2 and rhPrdx1 were determined by the SPR assay. KD values are shown above the traces. (**G**) Determination of Celastrol binding to rhPrdx2 protein by isothermal titration calorimetry (ITC). (**H**) Molecular interaction between Celastrol and Prxd2 as determined by docking software. Figure showing possible interaction between Celastrol and Cys172 of Prdx2. (**I, J**) Biotinylated-Celastrol interacts with Prdx2 in SGC-7901 cells and tumor tissues from mice implanted with SGC-7901 cells. Lysates were prepared from untreated SGC-7901 cells in culture and untreated mice subcutaneously injected with SGC-7901 cells. Biotinylated-Celastrol (Bio-Cel) was added to streptavidin-agarose beads. Biotin alone was used as a control. Lysates prepared from SGC-7901 cells (I) and tumor tissues from mice (J) were added. Eluent was then loaded on a polyacrylamide gels for immunoblotting. Total lysates were used as an input control. (**K**) Residue Cys172 of Prdx2 protein was mutated to Ala172 and Ser172. Prdx2 variants were probed for Celastrol interaction and compared to wildtype Prdx2, using pull-down assays described in panels I and J. (**L**) Peroxidase activity of rhPrdx2 proteins were monitored by measuring H_2_O_2_ levels as described in Methods (n = 3; *P<0.05, **P<0.01 compared to 0 µM control]. (**M, N**) rhPrdx2 proteins were incubated with Celastrol or Adenanthin for 10 min and peroxidase activity was measured (n = 3; *P<0.05, **P<0.01, ***P<0.001 compared to control). The IC_50_ of compounds against rhPrdx2 activity are shown. (**O**) Peroxidase activity following addition of Celasterol to rhPrdx1 (n = 3; *P<0.05, **P<0.01 compared to control). IC_50_ of Celastrol is shown. (**P**) Cellular Prdx enzyme activity was measured by adding Celastrol to lysate prepared from SGC-7901 cells (n = 3; *P<0.05, **P<0.01 compared to 0 µM control). The IC_50_ value of Celastrol was shown.

To understand how Celastrol induces ROS production and cancer cell apoptosis, we screened for potential proteins which bind to Celastrol. To do this, we used biotin-labeled Celastrol (bio-Celastrol) and assayed for the binding of Celastrol to recombinant proteins fabricated on HuProt^TM^ human protein microarray. Bio-Celastrol retains its biological activity as can be seen through viability tests (Figure S12). We reasoned that proteins binding to bio-Celastrol may represent direct targets of Celastrol and may contribute to its biological activity. We added bio-Celastrol to HuProt protein array (Figure 3A & B) and identified the top-ranking proteins as antioxidant peroxiredoxins (Prdx) (Figure 3C, Table S1). All Prdx 1-4 interacted with Celastrol, with Prdx2 generating the greatest signal-to-noise ratio (SNR) (Figure 3D, Table S1). Furthermore, assessment of other redox proteins showed low binding and underscored Prdx2 as the potential target of Celastrol (Figure S13). Interestingly, stable isotope labeling with amino acids in cell culture (SILAC) coupled to nanoLC-MS/MS analysis has recently shown Celastrol binding to Prdx1 in lymphoblastoid cell line 32 and human colon cancer line HCT116 33. Our studies and these recent findings suggest that Celastrol may potentially mediate its activities through binding and altering the activity of Prdx proteins.

We confirmed the binding of Prdx to Celastrol by additional assays including surface plasmon resonance (SPR) and isothermal titration calorimetry (ITC). SPR analysis at the molecular level showed that recombinant human (rh) Prdx2 binds to Celastrol with high affinity, yielding a KD of 5.5 µM (Figure 3E). In comparison, rhPrdx1, which produced a lower SNR in our protein array, showed a KD of 60 µM (Figure 3F). We then performed ITC and show that Celastrol bound to rhPrdx2 protein with a binding Kd value of 0.65 μM (Figure 3G). Molecular docking simulation shows that Cys172 is closest to Celastrol and suggests that Celastrol and Cys172 of Prdx2 may interact (Figure 3H). To confirm this Celastrol-Prdx interaction in intact systems, we performed pull-down assays with lysates from gastric cancer cell lines and tissues. Bio-Celastrol was added to streptavidin-agarose beads, and lysates from SGC-7901 cells as well as tumors generated in mice by implanting gastric cancer cells were applied. Eluent was then probed for Prdx2 by immunoblotting. Our results show that bio-Celastrol binds to Prdx2 protein in lysates from both gastric cancer cells in culture and gastric cancer tissues generated in mice (Figure 3I & J). These data indicate that Celastrol can directly bind to Prdx2 protein with high affinity.

As our molecular docking simulation showed proximity of Cys172 to Celastrol, we probed whether this residue participated in Celastrol binding. We performed site-directed mutagenesis to generate C172S and C172A variants and then subjected the variants to pull-down assays. Our results show that C172S and C172A reduce binding of Prdx2 with Celastrol (Figure 3K), indicating that this is a key residue in the interaction. To probe Prdx2-Celastrol interaction further, we tested whether ROS or Prdx2 activity alters the binding capacity. To test this, we performed the binding assay in the presence of H_2_O_2_, which serve the dual purpose of increasing ROS and activating Prdx2. We show that H_2_O_2_ reduces Prdx2 binding to Celastrol when introduced before Prdx2 and Celastrol have a chance to interact (Figure S14). This reduction is not seen when H_2_O_2_ is added after Prdx2-Celastrol brining.

As an antioxidant peroxidase, Prdx2 enzyme catalyzes the reduction of H_2_O_2_ to water in the presence of NADPH. We tested whether binding to Prdx2 to Celastrol inhibits the activity of Prdx2. We measured rhPrdx2 activity using Amplex Red-coupled spectrophotometric assay as described previously 21, 22 and show that increasing the concentration of rhPrdx2 reduces H_2_O_2_ in a cell-free system (Figure 3L). Importantly, presence of Celastrol supressed rhPrdx2 enzyme activity with an IC_50_ of 4.8 μM (Figure 3M). This inhibition was far greater than that mediated by adenanthin (Figure 3N), a known inhibitor of Prdx 23. Since our protein-Celastrol binding data showed interaction between Celastrol and other Prdx protein family members (Table S1, Table S2), we tested whether Celastrol also inhibits other Prdx proteins. Our results show that Celastrol inhibits Prdx1 activity but was found to be less effective against Prdx1 as compared to Prdx2 (Figure 3O). To confirm inhibition of Prdx activity by Celastrol in cells, we added Celastrol to SGC-7901 cell lysates and show reduced activity (Figure 3P). Collectively, these studies show that Celastrol directly binds to the antioxidant enzyme Prdx2 and inhibits its activity at both molecular and cellular levels.

### Overexpression of Prdx2 reduces Celastrol-induced gastric cancer cell apoptosis

**Figure 4:**
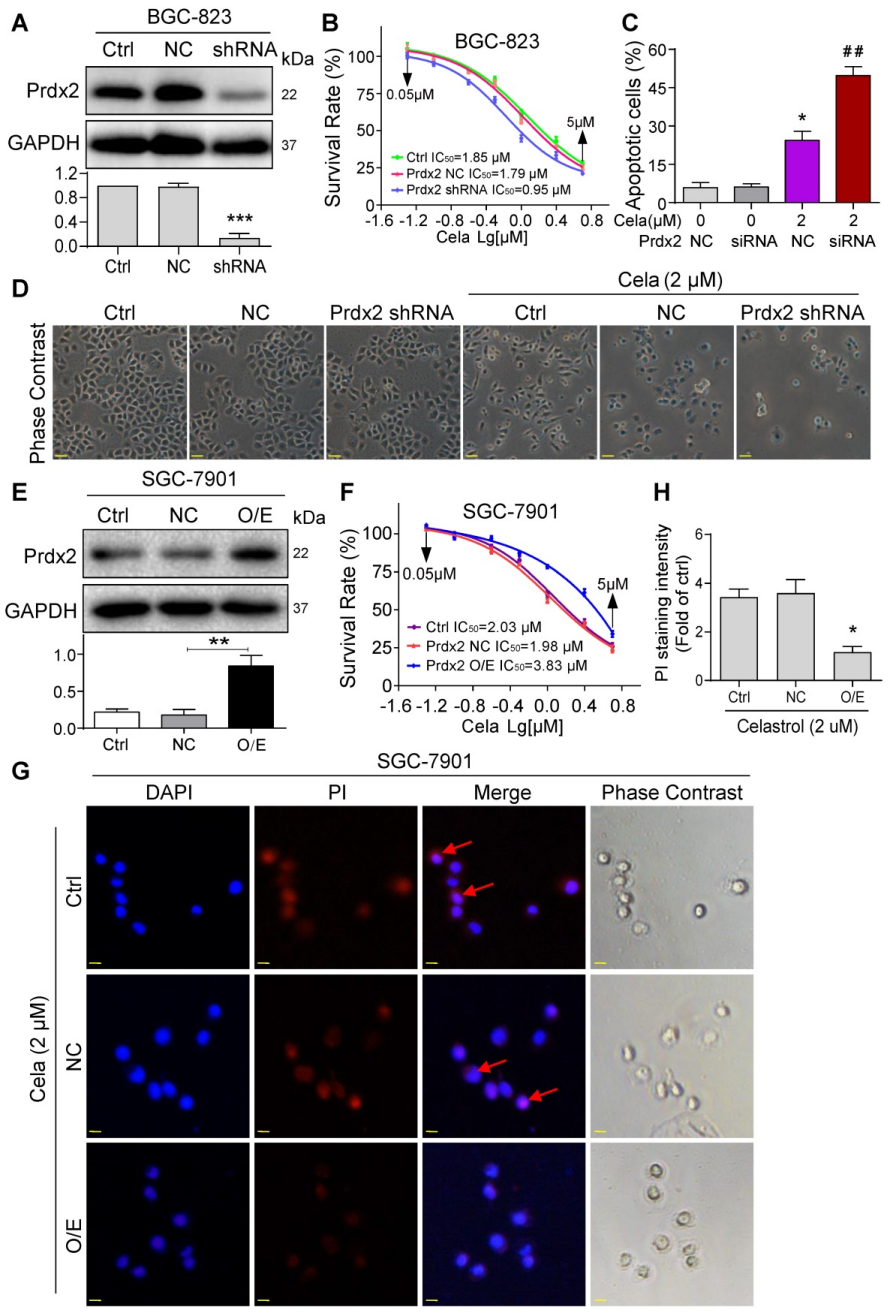
** Knockdown of Prdx2 potentiates and overexpression normalizes the activities of Celastrol.** (**A**) BGC-823 cells were stably transfected with negative control shRNA plasmid or shRNA targeting Prdx2. Total cell lysates were probed for Prdx2. GAPDH was used as loading control. Densitometric quantification is shown in lower panel. (**B**) Knockdown of Prdx2 in BGC-823 cells by shRNA transfection enhances Celastrol-induced cytotoxicity as determined by the MTT assay. (**C**) Knockdown of Prdx2 by siRNA transfection in BGC-823 cells significantly increased Celastrol-induced apoptotic cells. Cells were exposed to Celastrol at 2 µM for 24 h. Apoptosis was determined by Annexin/PI staining (siRNA = Prdx2 siRNA, NC = negative control siRNA; n = 3; *P<0.05, **P<0.01 compared to 0 µM Celastrol NC). (**D**) Representative phase contrast images of cells exposed to Celastrol for 24 h after Prdx2 knockdown (scale bar = 40 µm). (**E**) SGC-7901 cells were stably transfected with empty vector (NC) or Prdx2 cDNA (O/E). Lysates were probed for Prdx2 protein levels. GAPDH was used as loading control. Densitometric quantification is shown on right (n = 3; *P<0.05 compared to NC). (**F**) Prdx2 overexpressing cells and vector control transfected cells were exposed to Celastrol for 24 h. Cell viability was measured by MTT assay. IC_50_ values are shown. (**G**) PI staining of SGC-7901 cells showing reduced apoptosis (red; arrows) in cells expressing Prdx2 O/E plasmids. Cells were counterstained with DAPI (Blue) (scale bar = 10 µm). (**H**) The quantification of PI fluorescence intensity in cells expressing Prdx2 O/E plasmids (n = 3; *P<0.05 compared to NC).

We confirmed the involvement of Prdx2 in Celastrol-mediated gastric cancer cell death by modulating the levels of Prdx2. We assessed the relative levels of Prdx2 in the two cancer cell lines and show that BGC-823 cells express higher levels of Prdx2 compared to SGC-7901 (Figure S15). Based on this finding, we knocked down the expression of Prdx2 in BGC-823 cells (Figure 4A) and exposed the cells to Celastrol. Knockdown of Prdx2 decreased the IC_50_ of Celastrol by approximately 2x fold (Figure 4B). This decreased IC_50_ correlated with drastically increased number of apoptotic cells (Figure 4C & D). Conversely, overexpression of Prdx2 in SGC-7901 cells (Figure 4E), which initially expressed lower Prdx2 compared to BGC-823, increased the IC_50_ of Celastrol (Figure 4F) and reduced the level of Celastrol-induced apoptosis as determined by PI staining of cells (Figure 4G & H). These results show that Prdx2 deficiency potentiates the growth-inhibitory effects of Celastrol and overexpression partly rescues it.

### Celastrol inhibits gastric cancer organoids in culture and tumors in mice by increasing ROS and supressing Prdx

To explore whether Celastrol inhibits gastric cancer growth and whether such activities are associated with Prdx2 targeting, we first carried out modified organoid cultures. Briefly, small portions of human gastric cancer specimens were cultured *in vitro* in the presence of Celastrol for increasing time periods. Our results show that Celastrol limits the growth of gastric cancer tissues in culture (Figure S16A). This activity was associated with increased cleaved caspase 3 immunoreactivity (Figure S16B) and increased ROS levels as determined by DCFH-DA staining (Figure S16C). These findings provide supportive data that Celastrol inhibits gastric cancer growth through increased apoptosis and ROS induction.

**Figure 5:**
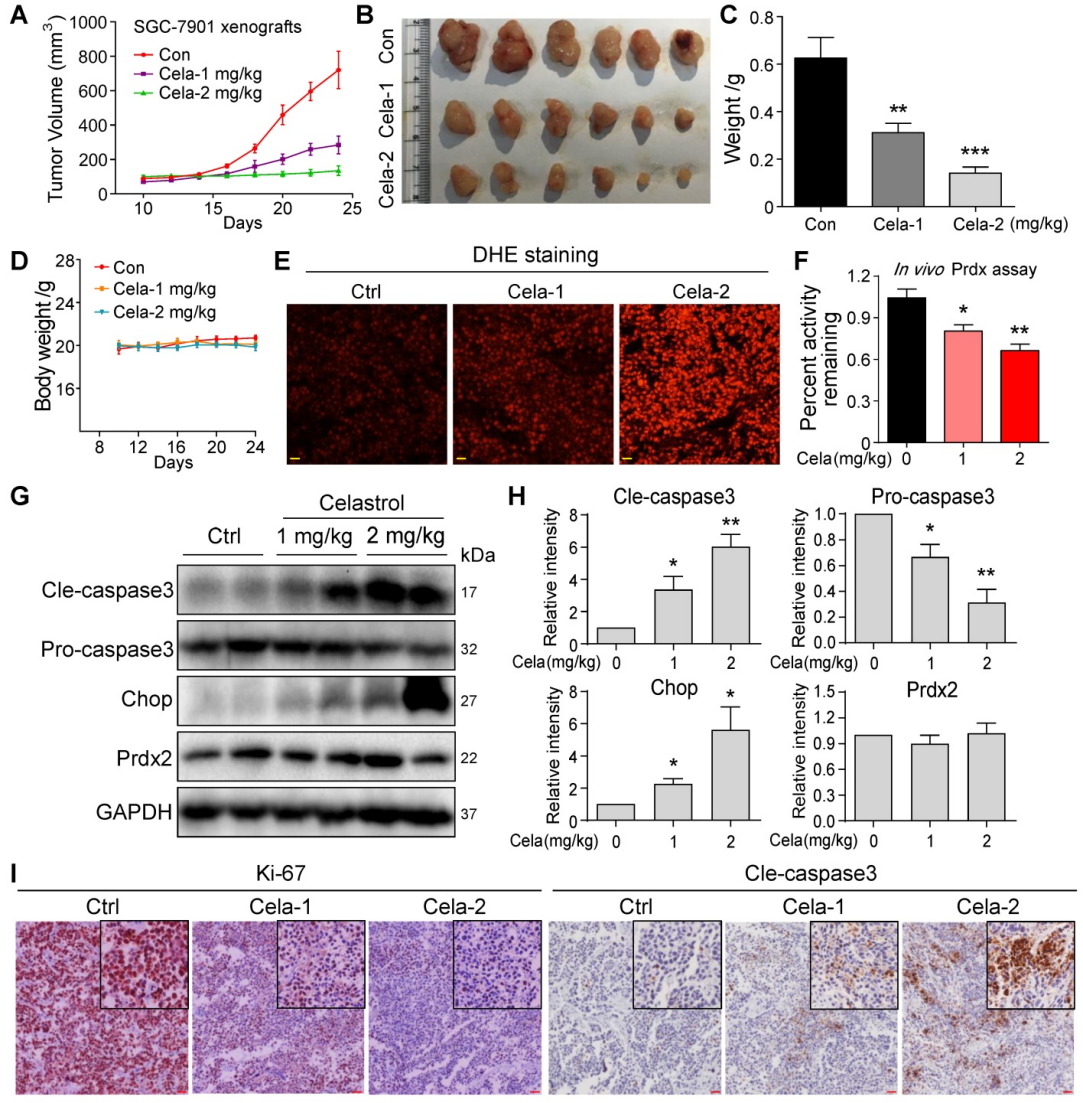
** Celastrol inhibits SGC-7901 xenograft tumor growth *in vivo* by decreasing Prdx enzyme activity and increasing ROS levels.** (**A-C**) Effect of Celastrol treatment on gastric cancer growth in mice. Figure showing tumor volumes (A), harvested tumors at 24-day follow-up (B), and tumor weights (C) (**P<0.01, ***P<0.001 compared to vehicle control). (**D**) Graph showing body weights of mice at indicated time points. (**E**) Staining of tumor tissue sections with ROS probe DHE (red). Increased fluorescence intensity is indicative of increased ROS levels (scale bar = 40 µm). (**F**) Prdx activity levels in harvested tumor specimens (*P<0.05, **P<0.01 compared to control). (**G**) Western blot analysis of cleaved-caspase3, pro-caspase3, Prdx2 and CHOP in tumor tissue lysates. GAPDH was used as protein loading control. (**H**) Quantification of cleaved-caspase3, pro-caspase3, Prdx2 and CHOP levels in tumor tissue lysates (n = 3; *P<0.05, **P<0.01 compared to control). (**I**) Immunohistochemical staining of tumor specimens for cell proliferation marker Ki-67 and apoptosis marker cleaved-caspase3. Immunoreactivity was detected by DAB chromogen (brown). Sections were counterstained with hematoxylin (blue). Inserts showing higher magnification (scale bar = 50 µm).

To build on our tissue culture studies, we injected SGC-7901 cells in mice and initiated Celastrol treatment at either 1 or 2 mg/kg. Follow-up at day 24 showed that Celastrol treatment reduces gastric tumor growth (Figure 5A-C). Even though 1 mg/kg dose was effective in reducing tumor growth, 2 mg/kg Celastrol completely prevented it. These effects were seen without changes to body weights of mice treated with Celastrol (Figure 5D) or histological assessment of pathological changes in heart, kidneys, and livers of mice (Figure S17). Analysis of harvested tumor specimens showed increased DHE staining indicative of elevated levels of ROS in Celastrol-treated mice (Figure 5E). Levels of malondialdehyde (MDA), products of polyunsaturated fatty acid peroxidation, were also increased by Celastrol, confirming increased oxidative stress (Figure S18). As identified in our *in vitro* studies, we measured the Prdx activity in tumor tissue lysates and show supressed levels upon treatment of mice with Celastrol (Figure 5F). The levels of Prdx2 proteins were not altered in tumor tissues (Figure 5G). These results support the notion that Celastrol binds to Prdx2 protein and inhibits its activity. Supressed Prdx activity and enhanced ROS levels by Celastrol correlated with increased levels of cleaved-caspase 3 and ER stress protein CHOP (Figure 5G & H), as well as reduced tumor cell ki-67 immunoreactivity (Figure 5I).

### ROS and Prdx2 mediate cancer-limiting activity of Celastrol in mice

**Figure 6:**
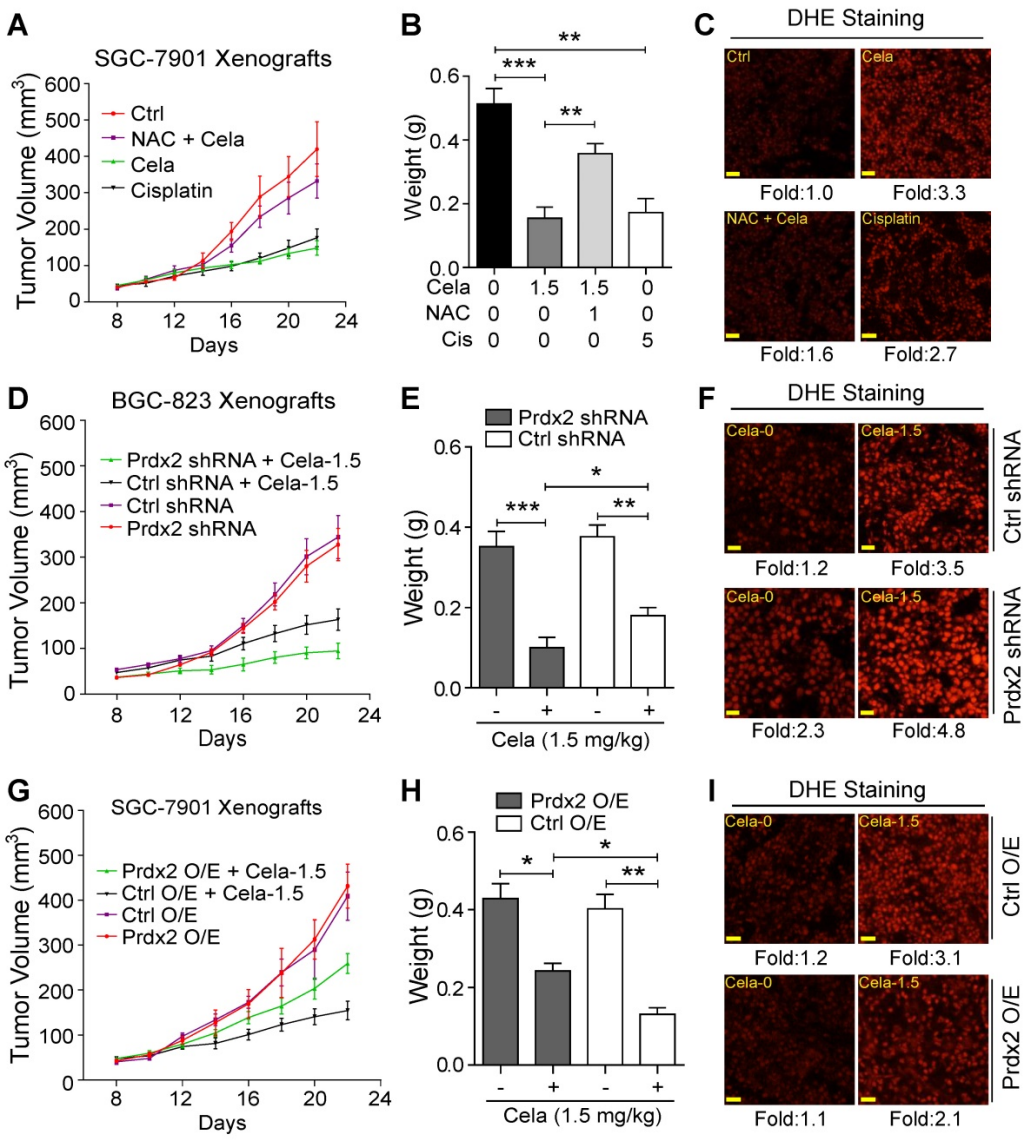
** ROS and Prdx2 mediate Celastrol-mediated inhibition of gastric cancer growth.** (**A**) Measurement of tumor volumes at indicated time points following implantation of SGC-7901 cells in BALB/c mice. Mice treated with 1.5 mg/kg Celastrol, NAC with 1.5 mg/kg Celastrol, or 5 mg/kg Cisplatin. NAC was given at 1 g/L in drinking water. Celastrol and Cisplatin treatments were administered every other day. Control mice received vehicle alone. (**B**) Tumor weights for mice in panel A (**P<0.01, ***P<0.001). (**C**) Staining of tumor tissue sections harvested from mice with ROS probe DHE (red) (scale bar = 50 µm). (**D**) Xenograft of Prdx2 shRNA-expressing BGC-823 cells. Mice were treated with 1.5 mg/kg Celastrol every other day (Ctrl shRNA = negative control shRNA). (**E**) Tumor weights for mice in panel D (*P<0.05, **P<0.01, ***P<0.001). (**F**) Staining of tumor tissue sections harvested from mice in panel D with ROS probe DHE (red) (scale bar = 50 µm). (**G**) Xenograft of Prdx2 cDNA expressing (O/E) SGC-7901 cells. Mice were treated with 1.5 mg/kg Celastrol every other day (Ctrl O/E = negative vector). (**H**) Tumor weights for mice in panel G (*P<0.05, **P<0.01). (**I**) Staining of tumor tissue sections harvested from mice in panel G with ROS probe DHE (red) (scale bar = 50 µm).

To determine whether the *in vivo* gastric cancer inhibitory activity of Celastrol is directly linked to the modulation of oxidative stress, mice were treated with NAC. For these studies we used 1.5 mg/kg Celastrol which is in the middle of the two effective doses in our experimental system. Similar to our initial studies, no histological abnormalities in heart, kidneys, and livers were noted in mice treated with 1.5 mg/kg Celastrol alone (Figure S19). Treatment of mice with NAC normalized the inhibitory activity of Celastrol on tumor growth (Figure 6A & B, Figure S20). DHE staining showed that NAC inhibited Celastrol-increased ROS level in tumor tissues (Figure 6C). Cisplatin, used as a positive control, increased ROS levels and reduced gastric cancer growth in mice.

We next explored the role of Prdx2 and injected mice with BGC-823 cells expressing Prdx2 shRNA. As mentioned, we selected BGC-823 cells for knockdown as these cells expressed higher level of Prdx2 protein compared to SGC-7901 cells. Prdx2 protein levels in the transfected cells remained low compared to control shRNA transfected cells for the duration of the *in vivo* studies (Figure S21). Our results show that Prdx2 shRNA-transfected cells grow significantly less compared to negative control shRNA transfected cells when mice are treated with Celastrol (Figure 6D & E, Figure S22). It is interesting to note that without Celastrol, no differences were found in tumors generated from control shRNA or Prdx2 shRNA transfected cells. In addition, Prdx2 knockdown showed increased DHE staining indicating elevation of ROS levels (Figure 6F). To strength these results, we overexpressed Prdx2 in SGC-7901 cells (Figure S23). Based on our cell culture studies, we expected a reversal of Celastrol activity in gastric cancer cells with Prdx2 overexpression (O/E). Indeed, overexpression of Prdx2 partially reversed the inhibitory effect of Celastrol on tumor growth (Figure 6G & H, Figure S24). As expected, DHE staining of tumors harvested from these mice showed reduced ROS levels upon overexpression of Prdx2 (Figure 6I). Together, these studies show that ROS and Prdx2 mediate the cancer growth-limiting activity of Celastrol in mice.

### Elevated Prdx2 in gastric cancer is associated with reduced survival

**Figure 7:**
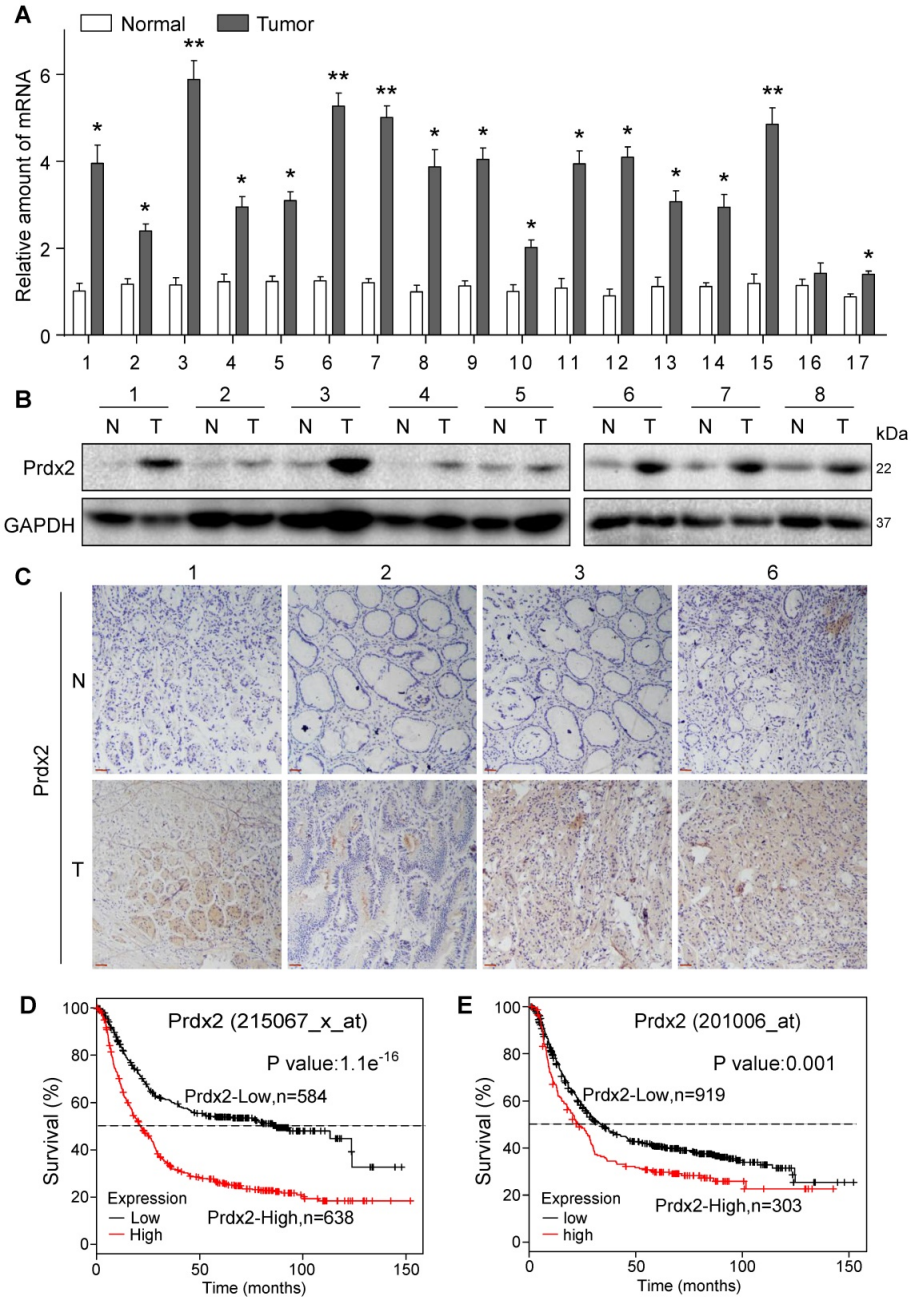
** Prdx2 is upregulated in human gastric cancer.** (**A**) Prdx2 mRNA levels in gastric cancer tissues (T) and patient-matched, tumor-adjacent normal gastric tissues (N). Transcript levels were normalized to β-actin levels. (**B**) Western blot analysis of Prdx2 protein levels in different gastric cancer tissues (T) and patient-matched, adjacent normal gastric tissues (N). GAPDH used as loading control. Data from the remaining 9 samples (n =17) is provided in Figure S25. (**C**) Representative immunohistochemical staining for Prdx2 (brown) in gastric cancer tissues (T) and adjacent normal gastric tissues (N). Slides were counterstained with hematoxylin (blue) [scale bar = 50 µm]. (**D**) Overall survival of the gastric cancer patients with high or low levels of Prdx2 using probe 215067_x_at. (**E**) Overall survival of the gastric cancer patients with high or low levels of Prdx2 using probe 201006_at.

To validate Prdx2 as a potential therapeutic target of gastric cancer, we assessed Prdx2 transcript levels in biopsy specimens obtained from patients with gastric cancer. Our results show that 15 of 17 samples analyzed had at least a 2x fold increase in Prdx2 in gastric cancer samples compared to patient-matched, tumor-adjacent samples (Figure 7A). The remaining 2 samples in our analysis showed approximately a 1.5× fold increase. Protein levels of Prdx2, measured by immunoblotting and tissue staining, also showed increased levels in cancer specimens compared to non-tumoral tissue (Figure 7B & C; Figure S25, Figure S26). These results show that Prdx2 is upregulated in gastric cancer and may play a role in its pathogenesis. To determine this, we used the Kaplan-Meier Plotter to stratify gastric cancer based on Prdx2 into high and low. Prdx2 probes included 215067_x_at (auto cutoff value used 265; Figure 7D) and 201006_at (auto cutoff value used 145; Figure 7E). Results show a strong association between high Prdx2 expression and low 5-year survival of patients (Figure 7D & E).

## Discussion

**Figure 8:**
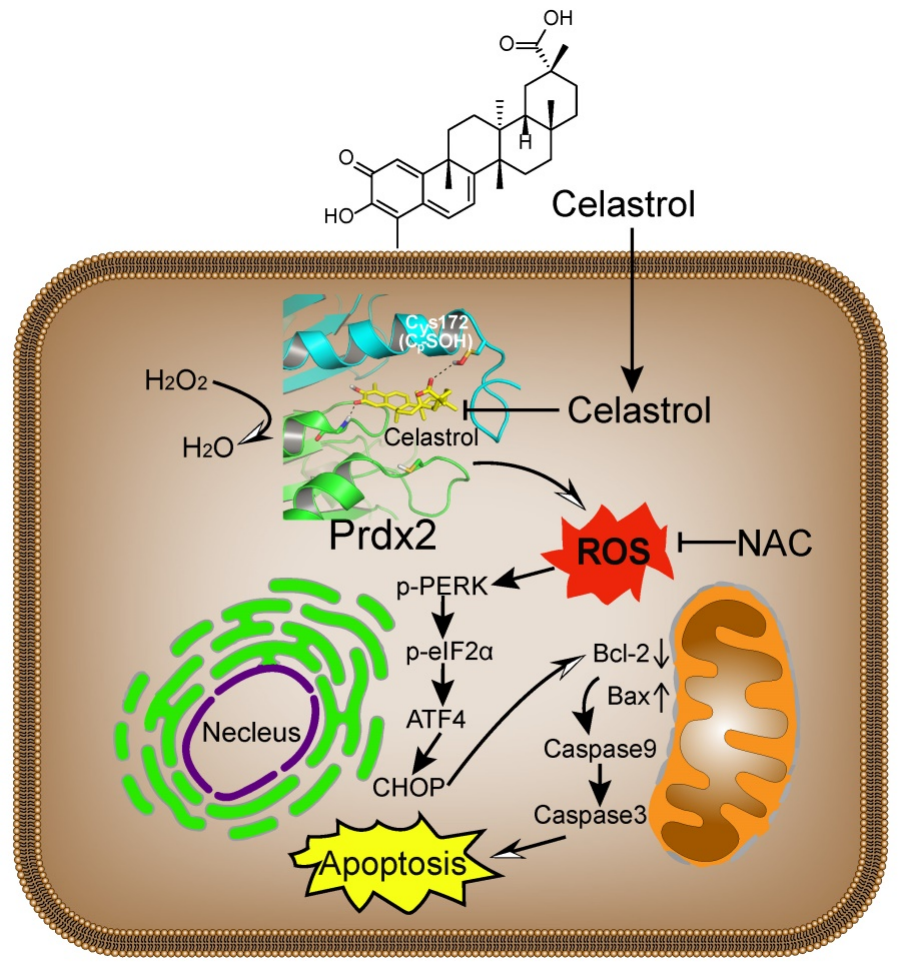
** Schematic illustration of the main findings of the study.** Celastrol binds to and inhibits Prdx2 in gastric cancer cells. Inhibition of Prdx2 augments ROS levels and initiates cell death through ER stress and mitochondrial dysfunction.

Broad interference of growth signals by natural compounds with pleiotropic actions may provide an opportunity to target gastric cancer effectively. Celastrol has recently been shown to provide growth-limiting activities in a panel of human cancers. Although changes in the activity of stress pathways such as JNK and nuclear factor-κB have been noted upon Celastrol treatment 16, 34, the mechanisms and targets underlying the inhibitory activity of Celastrol remain elusive. The key findings of our study, summarized in Figure 8, include the discovery that Celastrol limits gastric cancer growth by targeting Prdx2 and increasing ROS. Our detailed investigation shows that Celastrol enhances ROS generation in gastric cancer cells by inhibiting Prdx2, which in turn initiates ER stress and mitochondrial dysfunction and results in apoptotic cell death. In both culture and mouse model systems, antioxidant NAC prevented ROS increase and mitigated the growth-inhibitory activity of Celastrol. These findings provide support for Prdx2 and ROS modulators, such as Celastrol, as a potential treatment option for gastric cancer.

Using protein microarrays, we identified Prdx2 protein as a potential Celastrol-interacting protein. SPR and ITC analysis showed that Celastrol binds to recombinant Prdx2 protein. This interaction was further validated in pull-down and enzyme activity assays. It is important to note that Prdx2 is not the only target of Celastrol, as Celastrol appears to interact with multiple target proteins. In Prdx family alone, Celastrol interacted with Prdx1-4. Although, interaction with Prdx proteins 1, 3, and 4 was with lower affinity compared to Prdx2. Based on our results, Celastrol appears to function as a pan-Prdx inhibitor. This notion is supported by the modest rescue of cancer cell viability from Celastrol upon Prdx2 overexpression, but complete normalization by NAC. It is perhaps a safe assumption that Celastrol may have differential level of inhibition in gastric and other cancer cells, depending on the expression of Prdx proteins. In addition to Prdx proteins, Celastrol may also inhibit gastric cancer cells through targeting other proteins. For example, our protein arrays shows that Celastrol potentially interacts with signal transducer and activator of transcription (STAT), Alditol:NADP+ oxidoreductase, and heat shock protein 70 (HSP70). Recent studies do show that STAT 35, 36, aldose reductase 37, and HSP70 38 are linked to oxidative stress and cancer pathogenesis. Further studies are certainly warranted to determine the contribution of these potential Celastrol targets in gastric and other cancers.

One of the strongest links between Prdx dysregulation and cancer has come from knockout studies. Prdx1^+/-^ and Prdx1^-/-^ mice display a range of malignancies including lymphomas, hepatocellular carcinomas, osteosarcomas, islet cell adenomas, and adenocarcinomas of lung and breast 39. In contrast, deletion of Prdx2 in mice does not cause spontaneous neoplasia but is associated with hemolytic anemia 40. Furthermore, crossing Prdx2^-/-^ mice with H-rasG12V mice showed reduced hepatic tumor incidence when compared to Prdx2^+/+^ H-rasG12V mice 41. Silencing Prdx2 suppresses the growth of prostate cancer cells by inducing cell-cycle arrest at the G1 phase 42, and lung metastasis of melanoma cells 43. These studies show that Prdx2 may be involved in facilitating tumor progression and deficiencies in Prdx2 could help target cancer cells.

One mechanism by which cancer cells may survive elevated levels of ROS is through an overexpression of Prdx proteins 44, 45. Prdx2 expression is shown to be elevated in breast 46, lung 47, colorectal 48, and cervical 49 cancers compared to normal tissue. In colorectal cancer, increased expression of Prdx2 was found to be significantly associated with advanced local invasion, increased lymph node metastasis, and shorter disease-free survival 48. Prdx2 knockdown has also been shown to inhibit colorectal cancer growth, stimulate apoptosis, and augment the production of ROS 50. Previous studies in gastric cancer cells also show that depletion of Prdx2 reduces β-catenin levels and the expression of β-catenin-target genes 51. These alterations were associated with significantly reduced cell viability and invasive activity 51. Another study showed that transfection of gastric cancer cells with Prdx2 antisense plasmid enhanced cisplatin-induced cell death 52. These studies, together with our findings, show that Prdx2 inhibition may be a viable option for the management of gastric cancer.

One of the first reported Prdx inhibitors was adenanthin which promoted Prdx oxidation 53. Adenanthin induces differentiation in acute promyelocytic leukemia cells and shows efficacy in leukemia and hepatocellular carcinoma models 23, 54. Here, we report data that shows Celastrol as a Prdx inhibitor. It should be noted that clinical use of Celastrol is currently problematic because of a narrow therapeutic window and potential adverse effects. Although the dose of 2 mg/kg in our *in vivo* experiments did not cause any toxicity, higher doses may produce adverse effects as reported recently 55. However, Celastrol does represent a new lead compound for further design and discovery of Prdx inhibitors. Attempts at modifying Celastrol to reduce its toxicity, and using Celastrol in a targeted/controlled release format or in combinatorial therapies may help its clinical use.

In summary, we show that Celastrol effectively inhibits gastric cancer growth both *in vitro* and *in vivo* and may have therapeutic utility. We discovered that Prdx2 is one of the targets of Celastrol. Celastrol directly binds to and inhibits Prdx2 activity, which augments ROS accumulation to induce ER stress, mitochondrial dysfunction, and apoptosis in gastric cancer cells. Using human gastric cancer specimens and analyzing gene databases, we show that Prdx2 is overexpressed in gastric cancer. Therefore, the antioxidant Prdx2 protein may represent an excellent target to pursue for the development of therapies for gastric cancer patients.

## Supplementary Material

Supplementary figures and tables.

## Abbreviations

ATF4: activating transcription factor-4
Bax: Bcl-2-associated x protein
Bcl-2: B cell lymphoma-2
Cle-PARP: cleaved Poly (ADP-ribose) Polymerase
CHOP: CCAAT/enhancer-binding protein homologous protein
DCFH-DA: 2',7'-dichlorodihydrofluorescein diacetate
DHE: dihydroethidium
eIF2α: eukaryotic initiation factor 2
ER: endoplasmic reticulum
HSP70: heat shock protein 70
ip: intraperitoneal
ITC: isothermal titration calorimetry
MDA: malondialdehyde
NAC: N-acetyl-L-cysteine
NC: Negative Control
Nrf2: nuclear factor erythroid 2-related factor 2
PDB: Protein Data Bank
PI: propidium iodide
p-PERK: phosphorylated-protein kinase R (PKR)-like endoplasmic reticulum kinase
Prdx: peroxiredoxin
rh: recombinant human
ROS: reactive oxygen species
SNR: signal-to-noise ratio
SOD: superoxide dismutase
SPR: surface plasmon resonance
STAT: signal transducer and activator of transcription
Trx: thioredoxin
TrxR: thioredoxin reductase
