# Supplementary material for: Celastrol induces ROS-mediated apoptosis via directly targeting peroxiredoxin-2 in gastric cancer cells
Source: Theranostics. 2020 Aug 15;10(22):10290–308. doi: 10.7150/thno.46728 (PMC7481428; doi:10.7150/thno.46728)
Supplement: Supplementary file 1 — Supplementary figures and tables. [file thnov10p10290s1.pdf]

Supporting information

Celastrol induces ROS-mediated apoptosis via directly targeting peroxiredoxin-2 in gastric cancer cells

Xi Chen[1], Ying Zhao[1], Wu Luo[1], Sian Chen[2], Feng Lin[3], Xi Zhang[4], Shijie Fan[1], Xian Shen[2], Yi Wang[1]*, Guang Liang[1]*

[1] Chemical Biology Research Center, School of Pharmaceutical Science, Wenzhou Medical University, Wenzhou, Zhejiang, 325035, China
[2] Department of Gastrointestinal Surgery, the Second Affiliated Hospital of Wenzhou Medical University, Wenzhou, Zhejiang, 325000, China
[3] Department of Gynaecology, the First Affiliated Hospital of Wenzhou Medical University, Wenzhou, Zhejiang, 325035, China
[4] The Affiliated Yueqing Hospital, Wenzhou Medical University, Wenzhou, Zhejiang, 325600, China

Short title: Celastrol limits gastric cancer by targeting Prdx2

Corresponding author:

Yi Wang, Ph.D, Associate professor
Chemical Biology Research Center, School of Pharmaceutical Sciences, Wenzhou Medical University, Wenzhou325035, China; E-mail: yi.wang1122@wmu.edu.cn; Phone number: +86-577-86699396
and
Guang Liang, PhD, Professor
Chemical Biology Research Center, School of Pharmaceutical Sciences, Wenzhou Medical University, Wenzhou325035, China; E-mail: wzmcliangguang@163.com

Contents
Supplemental information includes 4 tables and 26 figures.

Table S1: The Signal-to-noise ratio (SNR) of Celastrol binding protein clusters.

Category	Protein	SNR
Peroxiredoxin, C-terminal	Prdx2	6.05
	Prdx1	5.25
	Prdx4	4.04
	Prdx3	3.11
Peroxiredoxin, AhpC-type	Prdx2	6.05
	Prdx1	5.25
	Prdx4	4.04
VHS	TOM1L2	6.07
	TOM1	4.32
	HGS	3.79
	GGA1	3.56
	GGA3	5.03
STATs	STAT4	3.44
	STAT5A	3.21
	STAT3	3.95
domain:UBA 1	RAD23A	4.68
	USP5	3.21
	UBAP1	3.16
$NADP^+$ -oxidoreductase activity	AKR1B1	4.33
	AKR7A2	3.31
	AKR1C1	3.93
Alpha_adaptinC2	AP1B1	3.21
	GGA1	3.56
	GGA3	5.03
HSP70, conserved site	HSPA2	3.2
	HSPA6	8.89
	HSPA1A	4.45
	HSPA8	3.98
PAPS metabolic process	SULT1A1	4.32
	SULT2B1	3.05
	SULT1C2	3.02

Table S2: The Signal-to-noise ratio (SNR) of Celastrol-binding Prdxs protein.

Bio-Celastrol	SNR
Prdx2	6.05
Prdx1	5.25
Prdx4	4.04
Prdx3	3.11
Prdx5	1.04
Prdx6	0

Table S3: Clinical characteristics of gastric cancer patients. Chart is based on TNM staging of UICC (International Union Against Cancer) eighth edition of Malignant Tumors.MAC Stage is improved Astler-Coller staging.

Number	Sex	Age	Tumor size (cm)	Depth of invasion	Lymph node metastasis	Differentiation status	TNM stage	MAC stage
1	Male	73	6.0×3.0	T4a	N1b	Poor-Moderate differentiation	IIB	B2
2	Female	69	4.0×3.0	T4a	N0	Moderate differentiation	IIC	B3
3	Female	71	4.5×3.5	T4a	N2b	Poor differentiation	IIIB	C2
4	Male	65	3.0×3.0	T2	N0	Moderate-well differentiation	IIA	B2
5	Female	72	0.8×2.5	T3	N0	Moderate-well differentiation	IIA	B2
6	Male	66	3.0×2.0	T3	N0	Moderate differentiation	IIB	B2
7	Male	68	3.5×2.8	T2	No	Moderate-well differentiation	IIA	B2
8	Male	49	5.0×4.5	T3	N1a	Poor-Moderate differentiation	IIIA	C2
9	Male	72	4.0×3.5	T4a	N0	Poor-Moderate differentiation	IIIB	C2
10	Female	70	2.0×1.5	T4b	N0	Moderate-well differentiation	I	A
11	Female	56	5.0×5.0	T4a	N21	Poor differentiation	IIA	B2
12	Male	57	8.0×6.0	T4b	N2b	Poor-Moderate differentiation	IVC	/
13	Male	61	2.8×0.6	T4a	N0	Moderate differentiation	IIIA	C2
14	Male	59	5.0×4.0	T4b	N2a	Poor differentiation	IIIC	C3
15	Female	64	3.5×3.0	T3	N1b	Moderate differentiation	IIIA	C1
16	Male	60	4.5×2.8	T4b	N0	Moderate differentiation	IIIB	C2
17	Male	68	3.0×2.5	T4a	N0	Moderate differentiation	IIIA	C1

Table S4. Primers used in this study.

Prdx2_F	CCAGACGCTTGTCTGAGGAT
Prdx2_R	ACGTTGGGCTTAATCGTGTC
β-actin_F	AGCCTCGCCTTTGCCGATCCG
β-actin_R	TCTCTTGCTCTGGGCCTCGTCG
Prdx2_C172S_F	GGGGGAAACTTCCCCATGCTCGTCTGTGTACTG
Prdx2_C172S_R	GCTGGCTGGAAGCCTGGCAGTGACACGAT
Prdx2_C172A_F	GGGGGCAACTTCCCCATGCTCGTCTGTGTACTG
Prdx2_C172A_R	GCTGGCTGGAAGCCTGGCAGTGACACGAT

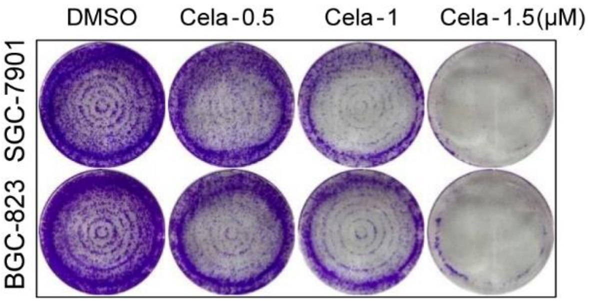

Figure S1: Effect of varying Celastrol concentrations on gastric cancer cell colony formation. Cells were incubated with Celastrol (Cela) for 8 h and allowed to grow for 8 days in normal growth media. Colonies were stained by crystal violet dye (blue).

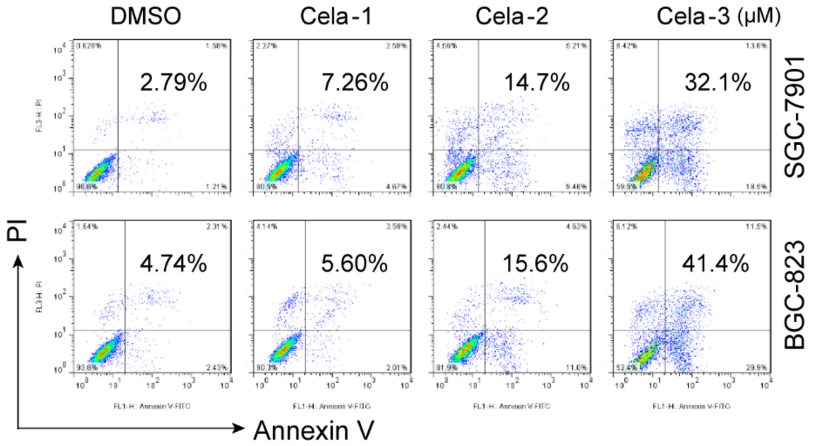

Figure S2: Effect of Celastrol on gastric cancer cell apoptosis. Cells were exposed to Celastrol (Cela) for 24 h. Apoptotic cells were assessed by Annexin V/PI staining and flow cytometric quantification of double-positive cells. Representative flow cytometry graphs are shown here. Bar graphs are shown in Figure 1C.

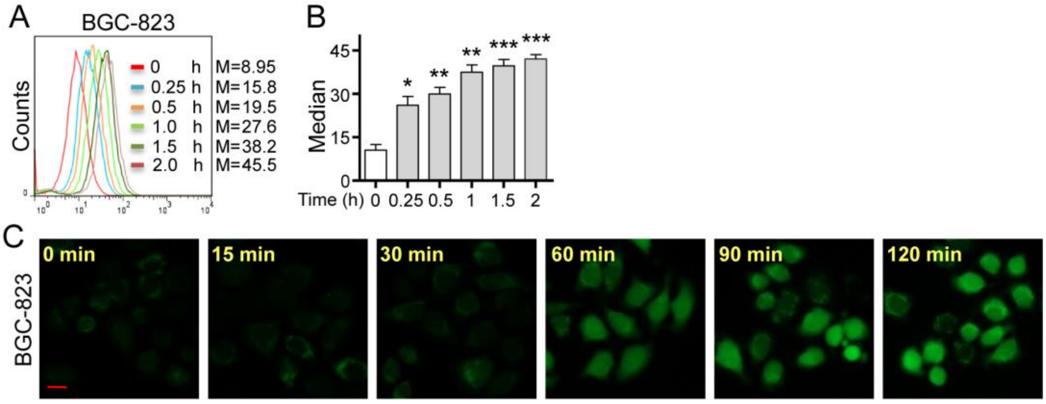

Figure S3: Celastrol induces ROS in BGC-823 cells. **(A, B)** ROS generation was measured by DCFH-DA staining. BGC-823 cells were challenged with 3 µM Celastrol for indicated times. Representative flow cytometric graph shown in panel A. Quantification of ROS levels in SGC-7901 cells is shown in panel B [n = 3; *P<0.05, **P< 0.01, ***P<0.001 compared to 0 h control]. **(C)** Fluorescence images of DCFH-DA-stained cells (green) were captured [scale bar = 20 µm].

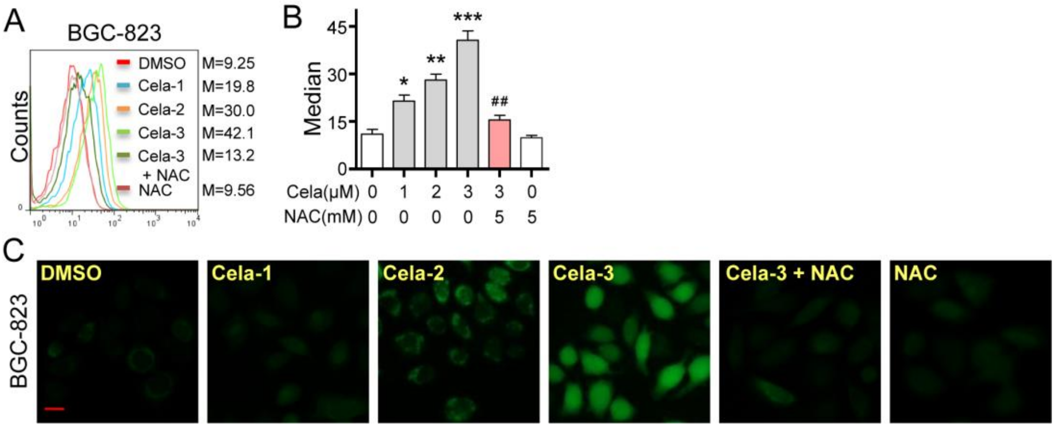

Figure S4: NAC reduces Celastrol-induced ROS. **(A, B)** BGC-823 cells were challenged with Celastrol at 1, 2, or 3 µM for 3 h. NAC was used at 5 mM, either alone or as a 1 h pretreatment. Representative flow cytometric graph shown in panel A. Quantification of ROS levels in BGC-823 cells is shown in panel D [n = 3; *P<0.05, **P< 0.01, ***P<0.001 compared to Control; ##P<0.01 compared to Cela-3]. **(C)** BGC-823 cells were treated as indicated in panel A and fluorescence images showing DCFH-DA (green) were captured [scale bar = 20 µm].

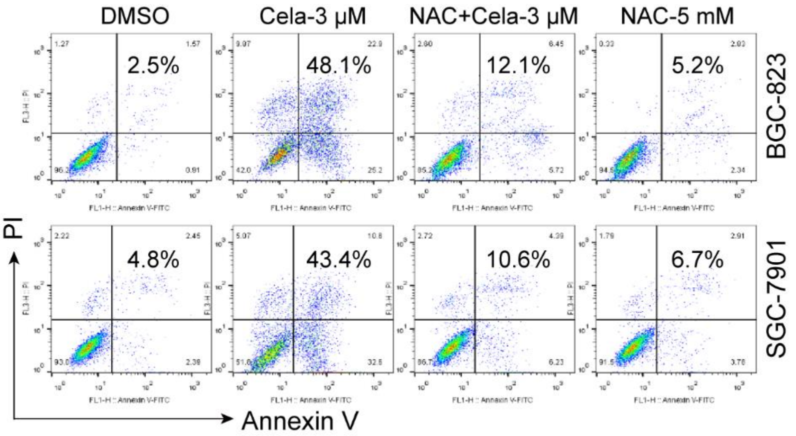

Figure S5: Effect of NAC on Celastrol-induced apoptosis. SGC-7901 and BGC-823 cancer cell lines were pretreated with 5mM NAC for 1h and then exposed to 3μM Celastrol for 24 h. Apoptotic cells were assessed by Annexin V/PI staining. Representative flow cytometry graphs are shown here. Quantification of apoptotic cells is shown in Figure 1J.

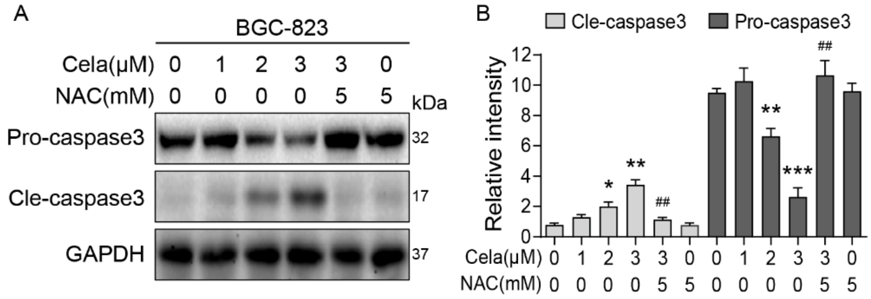

Figure S6: NAC inhibits Celastrol-induced caspase-3 activation. BGC-823 cells were exposed to Celastrol at indicated concentrations for 20 h, with or without 1 h pretreatment with 5 mM NAC. Western blot analysis of the cell lysates for cleaved-caspase-3 and caspase-3 was performed. with GAPDH was used as loading control. Panel A shows immunoblots of cleaved-caspase-3 and caspase-3. GAPDH used as loading control. Panel B shows quantification of cleaved-caspase-3 and caspase-3 levels in SGC-7901 cells [n = 3; *P<0.05, **P< 0.01, ***P<0.001 compared to Control; ##P<0.01 compared to Cela-3].

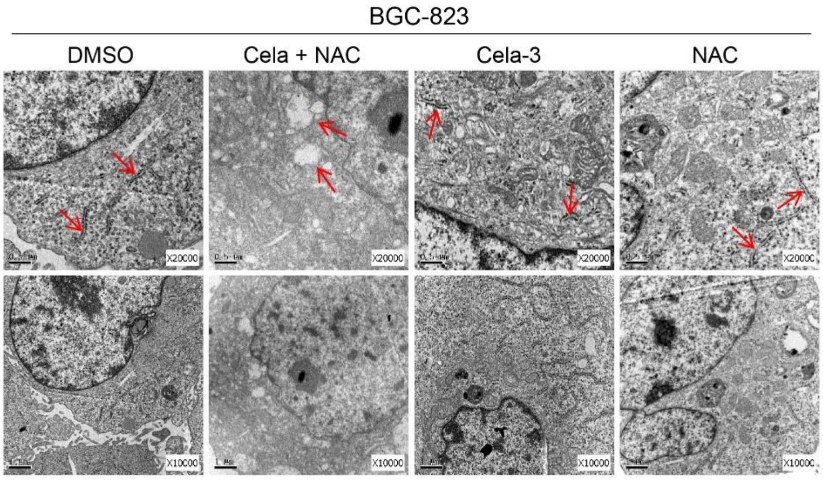

Figure S7: Celastrol activates ROS-mediated ER stress in BGC-823 cells. Electron microscopy images of BGC-823 cells exposed to 3 μM Celastrol for 8 h. Arrows pointing to ER [images shown are 10000 magnification in lower panel and 20000 magnification in upper panels].

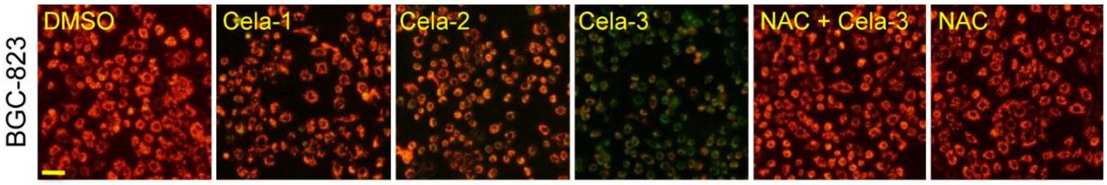

Figure S8: Mitochondrial membrane potential disruption by Celastrol. Mitochondrial membrane potential (Δψm) was detected by JC-1 dye. BGC-823 cells were pretreated with 5 mM NAC for 1 h before exposure to 3 μM Celastrol for 12 h. Cells were then stained with JC-1 dye and fluorescence images were captured [scale bar = 40 μm].

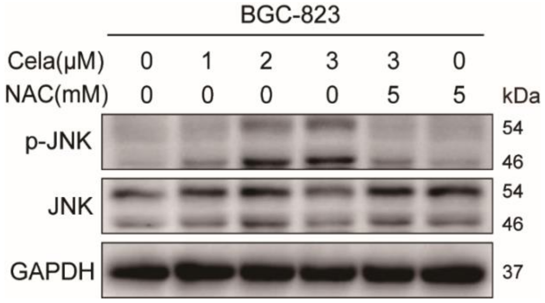

Figure S9: NAC blocked Celastrol-induced JNK activation. Western blot analysis of p-JNK/JNK in BGC-823 cells exposed to Celastrol at indicated concentrations for 12 h. NAC pretreatment was carried out at 5 mM for 1 h. GAPDH was used as loading control.

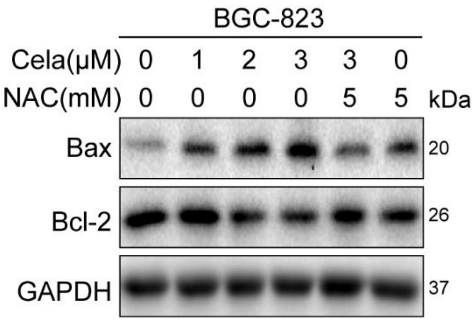

Figure S10: NAC blocked Celastrol-induced cell survial proteins. Western blot analysis of Bax/Bcl-2 in BGC-823 cells exposed to Celastrol at indicated concentrations for 12 h. NAC pretreatment was carried out at 5 mM for 1 h. G
APDH was used as loading control.

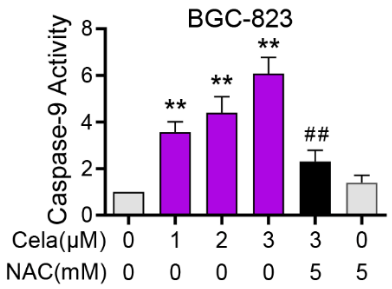

Figure S11: NAC inhibits Celastrol-induced caspase-9 activation. BGC-823 cells were exposed to Celastrol at indicated concentrations for 20 h, with or without 1 h pretreatment with 5 mM NAC. Caspase-9 activity was measured using a substrate kit [n = 3; **P<0.01 compared to Control; ##P<0.01 compared to Cela-3].

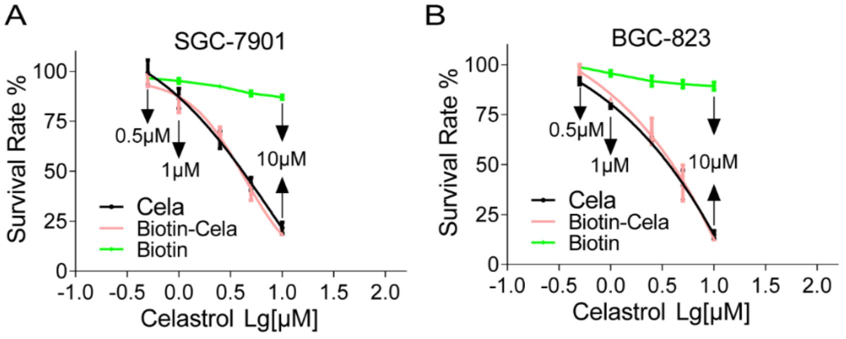

Figure S12: Biotinylated-Celastrol retains cytotoxic activity against gastric cancer cells. SGC-7901 **(A)** and BGC-823 cells **(B)** were exposed to Celastrol, biotinylated-Celastrol, or free biotin at increasing concentrations for 24 hours. Cell viability was measured by the MTT assay.

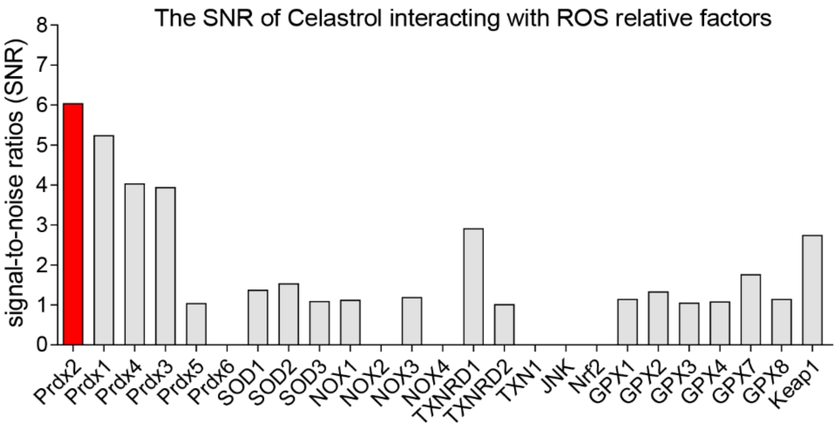

Figure S13: The signal-to-noise ratios (SNR) of Celastrol interacting with ROS-associated proteins.

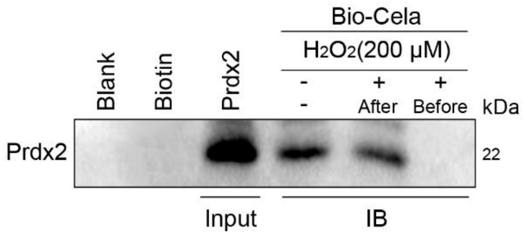

Figure S14: Effect of H_2O_2 on the interaction between Prdx2 and Celastrol. lane 1 (blank), lane 2 (free biotin), lane 3 (rhPrdx2), lane 4 (Bio-Cela with rhPrdx2 (IB) but no H_2O_2), lane 5 (Bio-Cela with rhPrdx2 for 4 h before H_2O_2 treatment), lane 6 (rhPrdx2 was treatment by H_2O_2 for 2 h, then allowed to interact with Bio-Cela).

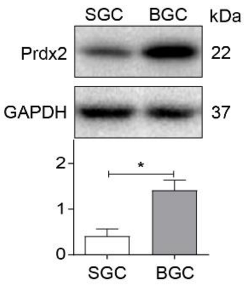

Figure S15. Levels of Prdx2 protein in SGC-7901 and BGC-823 cells as measured by western blot. GAPDH was used as loading control. Quantification is shown in lower panel [*P<0.05].

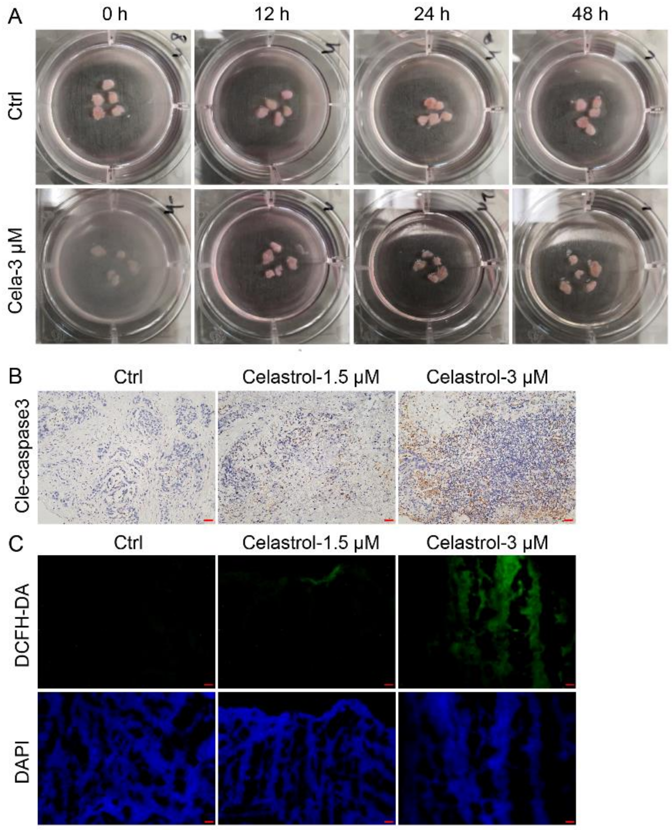

Figure S16: The effect of Celastrol on human gastric cancer specimens in culture. **(A)** Image showing culture of human gastric cancers in 3D culture system. **(B)** Cleaved caspase3 immunostaining of tumor specimens following Celastrol exposure. Immunoreactivity was detected by diaminobenzidine (brown). Samples were counterstained with hematoxylin (blue) [scale bar = 100 μm]. **(C)** ROS levels in tumor tissues as detected by DCFH-DA staining (green). Images were captured using epifluorescence microscope [scale bar = 100 μm].

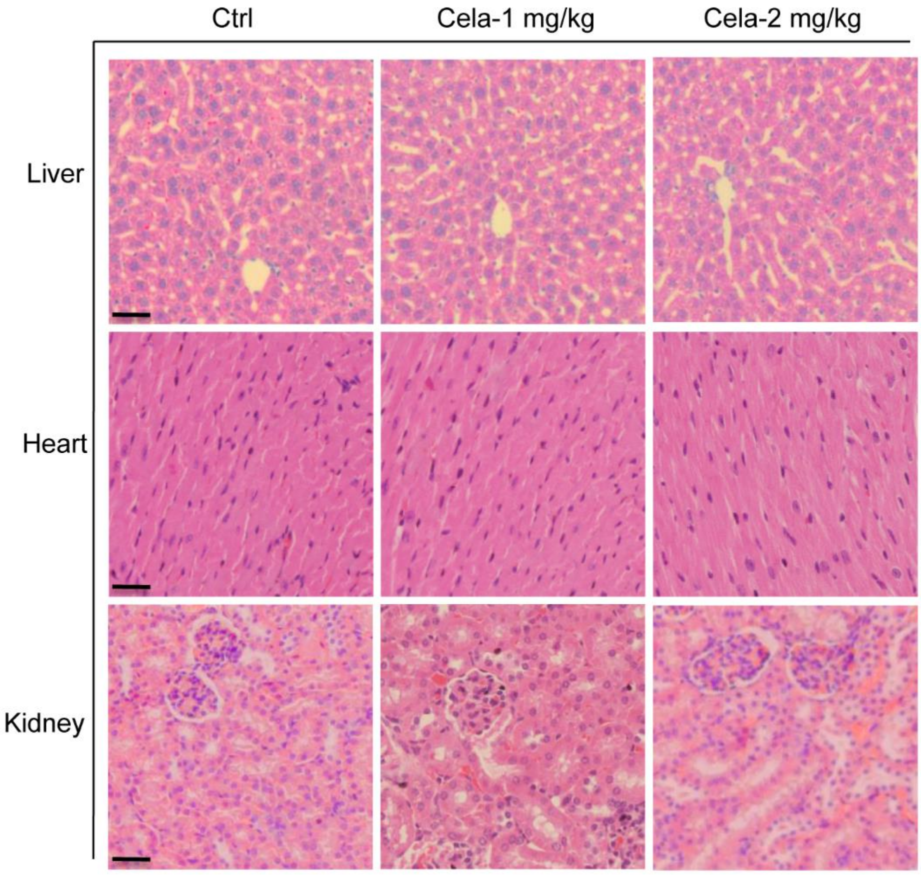

Figure S17: Effect of intraperitoneal Celastrol administration on various organs in mice. Hematoxylin and eosin staining of liver, kidney, and heart tissues from mice treated with 1 mg/kg or 2 mg/kg Celastrol. Treatments were carried out every other day for 24 days. No pathological alterations were noted upon Celastrol treatment [scale bar = 50 μm].

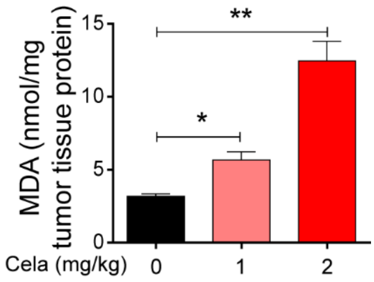

Figure S18: Celastrol induces oxidative stress in gastric tumors in mice. Levels of malondialdehyde (MDA), a product of lipid peroxidation, in tumors harvested from mice implanted with SGC-7901 cells. Mice were treated with 1 mg/kg or 2 mg/kg Celastrol every other day for 24 days [*P<0.05, **P<0.01 compared to vehicle control].

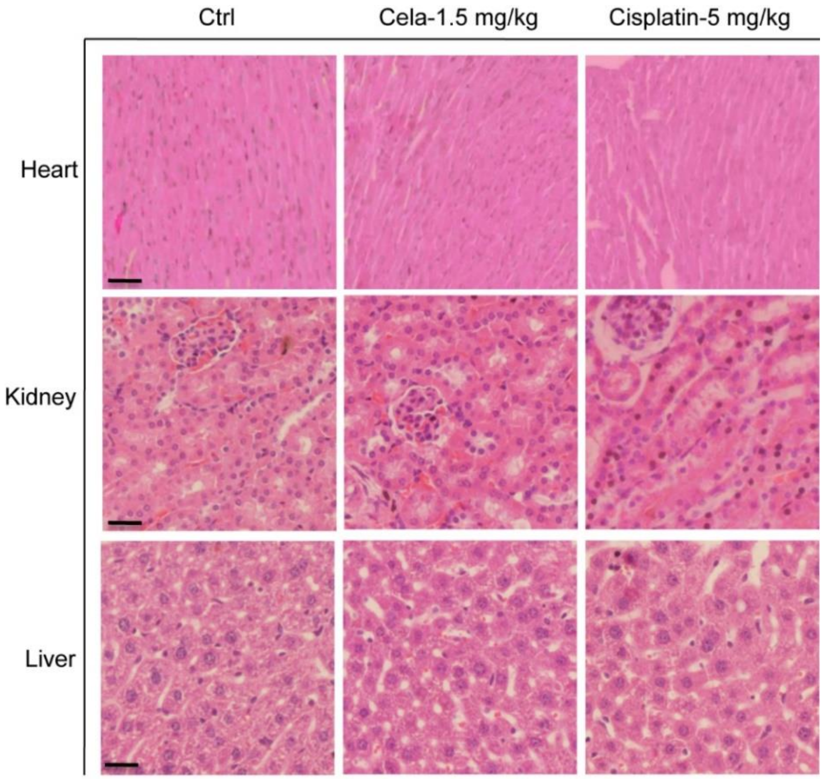

Figure S19: Effect of intraperitoneal Celastrol and Cisplatin on various organs in mice. Hematoxylin and eosin staining of liver, kidney, and heart tissues from mice treated with 1.5 mg/kg Celastrol or 5 mg/kg Cisplatin every other day for 24 days [scale bar = 50 μm].

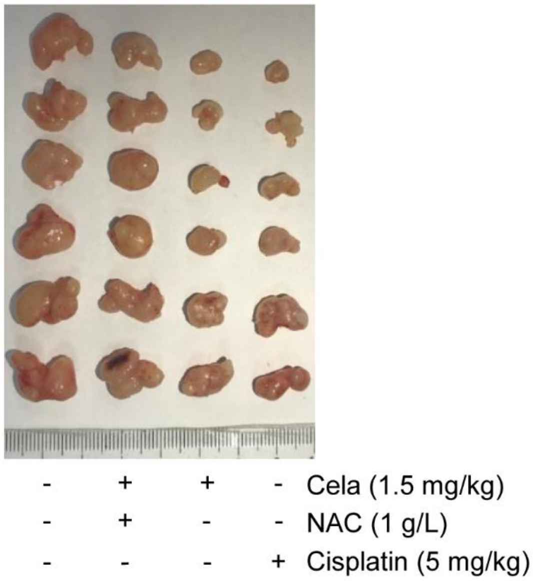

Figure S20: NAC prevents Celastrol-induced gastric tumor growth in mice. Images of harvested tumors from mice. SGC-7901 cells were injected in BALB/c mice. Mice were then divided into four experimental groups: 1) vehicle control group, 2) 1.5 mg/kg Celastrol every other day, 3) 1.5 mg/kg Celastrol every other day and given 1 g/L NAC in drinking water, and 4) 5 mg/kg Cisplatin every other day.

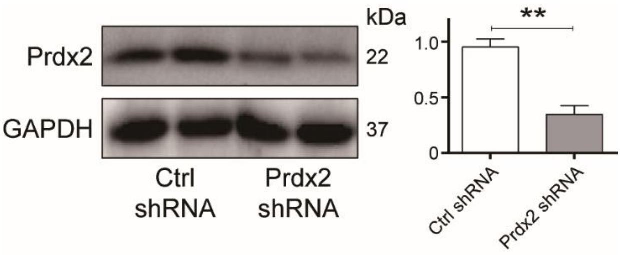

Figure S21: Prdx2 levels in tumors produced from Prdx2-shRNA expressing gastric cancer cells. Mice were injected with BGC-823 cells expressing Prdx2 shRNA or negative control shRNA (Ctrl shRNA). Tumors were harvested after 24 days and protein levels were determined by immunoblotting. GAPDH was used as loading control. Densitometric quantification is shown on right [n = 3; **P< 0.01 compared to Ctrl shRNA].

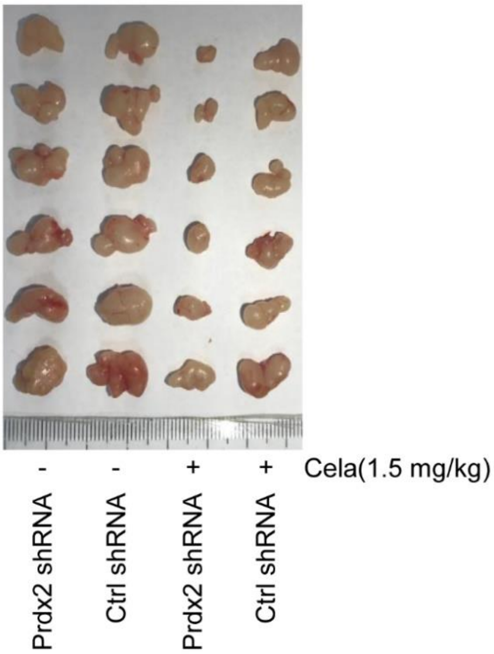

Figure S22: Prdx2 knockdown enhances the *in vivo* inhibitory activity of Celastrol. Images of harvested tumors from mice. BGC-823 cells transfected with negative control shRNA or shRNA targeting Prdx2 were injected in mice. Mice were then treated with 1.5 mg/kg Celastrol every other day for 24 days.

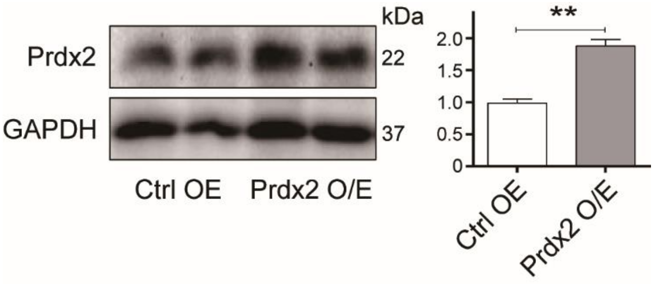

Figure S23: Prdx2 levels in tumors produced from Prdx2 overexpressing gastric cancer cells. Mice were injected with SGC-7901 cells expressing Prdx2 cDNA (Prdx2 O/E) or empty vector (Ctrl O/E). Tumors were harvested after 24 days and protein levels were measured by immunoblotting. GAPDH was used as loading control. Densitometric quantification is shown on right [n = 3; **P< 0.01 compared to Ctrl O/E].

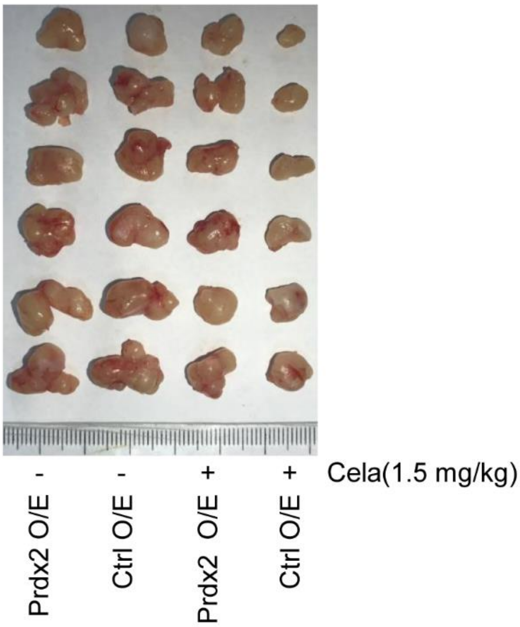

Figure S24: Prdx2 overexpression reduces the *in vivo* inhibitor activity of Celastrol. Images of harvested tumors from mice. SGC-7901 cells transfected with empty control vector or Prdx2 cDNA were injected in mice. Mice were then treated with 1.5 mg/kg Celastrol every other day for 24 days.

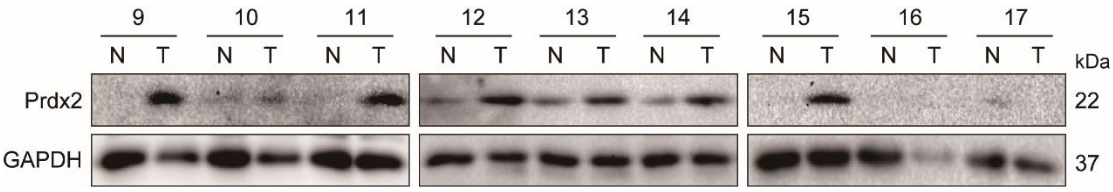

Figure S25: Prdx2 protein levels in human gastric cancer specimens. Human gastric tumor specimens (T) and patient-matched, tumor-adjacent tissues (N) were probed for Prdx2 protein levels by western blotting. GAPDH was used as loading control. Figure showing samples 9-17. Remaining samples (1-8) are included in Figure 7D.

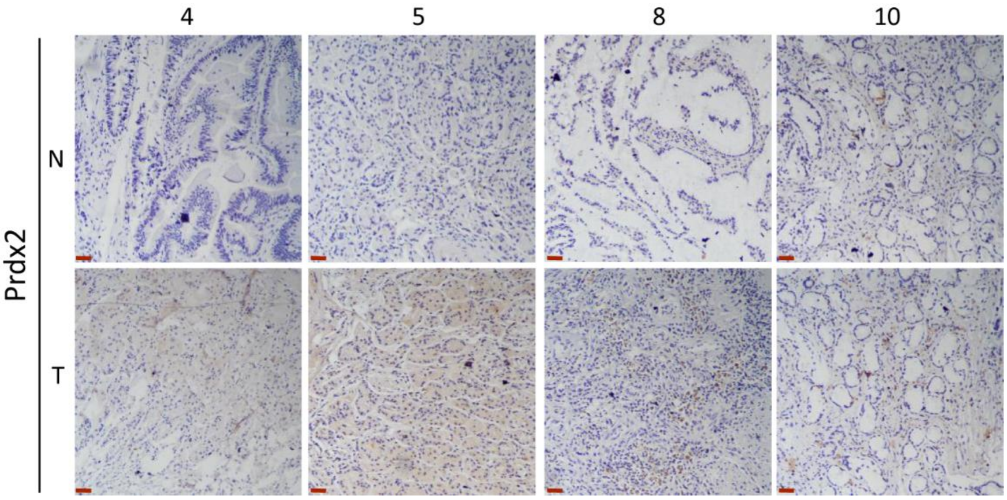

Figure S26: Prdx2 immunostaining of human gastric cancer specimens. Immunohistochemical staining for Prdx2 in human gastric cancer tissues (T) and patient-matched, tumor-adjacent tissues (N). Staining was visualized by diaminobenzidine (brown). Slides were counterstained with hematoxylin (blue). Numbers indicate deidentified patient samples [scale bar = 100 μm].
